# Targeting Oncofoetal Chondroitin Sulphate Allows Identification of Tumour‐Derived Extracellular Vesicles

**DOI:** 10.1002/jev2.70106

**Published:** 2025-06-17

**Authors:** Agustin Enciso‐Martinez, Caroline Løppke, Joyce J. Koene, Jade Ebbelaar, Meike van der Geest, Mandy Los, Emma P. E. M. Boerrigter, Robert Dagil, Tobias Gustavsson, Elena E. Vidal‐Calvo, Ton G. van Leeuwen, Roman I. Koning, Nick van Es, Ali Salanti, Edwin van der Pol, Rienk Nieuwland, Mette Ø. Agerbæk, Peter ten Dijke

**Affiliations:** ^1^ Oncode Institute Department of Cell and Chemical Biology Leiden University Medical Center Leiden the Netherlands; ^2^ Amsterdam UMC, University of Amsterdam Biomedical Engineering & Physics, Meibergdreef 9; Cancer Center Amsterdam, Imaging and Biomarkers University of Amsterdam Amsterdam the Netherlands; ^3^ Amsterdam UMC, Laboratory of Experimental Clinical Chemistry, Laboratory Specialized Diagnostics & Research, Department of Laboratory Medicine University of Amsterdam Amsterdam the Netherlands; ^4^ Centre for translational Medicine and Parasitology Department of Immunology and Microbiology Faculty of Health and Medical Sciences University of Copenhagen Copenhagen Denmark; ^5^ VAR2 Pharmaceuticals ApS Frederiksberg Denmark; ^6^ Electron Microscopy Facility Department of Cell and Chemical Biology Leiden University Medical Center Leiden the Netherlands; ^7^ Department of Vascular Medicine Amsterdam UMC University of Amsterdam Amsterdam the Netherlands; ^8^ Amsterdam Cardiovascular Sciences, Pulmonary Hypertension & Thrombosis Amsterdam the Netherlands; ^9^ VarCT Diagnostics Frederiksberg Denmark

**Keywords:** cancer, extracellular vesicles, liquid biopsy, pancreatic adenocarcinoma, VAR2CSA, biomarker

## Abstract

Tumour‐derived extracellular vesicles (tdEVs) are widely studied for their contribution to tumour progression and metastasis. These studies are hampered by the lack of specific markers to identify the tdEVs. Here, we show that oncofoetal chondroitin sulphate, a malignancy‐associated glycosaminoglycan modification, is present on tdEVs and can be targeted by the malaria VAR2CSA protein or C9 antibody. Using a fluorescently labelled recombinant VAR2CSA protein, we identified EVs from cancer cells in vitro by super‐resolution fluorescence microscopy and flow cytometry, as well as in a proof‐of‐concept study using plasma samples from pancreatic adenocarcinoma patients. Thus, the binding of VAR2CSA offers a tool to identify tdEVs, and can be used to explore their function and biomarker potential in cancer.

## 1 Introduction

Extracellular vesicles (EVs) are membrane‐delimited particles released by all cells. Increasing evidence suggests that tumour‐derived EVs (tdEVs) contribute to organ‐specific metastasis and disease progression (Hoshino et al. 2015, Peinado et al. 2012). Moreover, EVs have gained attention for their potential as biomarkers from liquid biopsies and as delivery vehicles for therapeutic agents (Andre et al. 2024; Du et al. 2023; Kumar et al. 2024).

Despite the growing interest in circulating tdEVs, their detection and analysis are challenging due to multiple factors. EVs, and particularly tdEVs in blood, are outnumbered by other particles, while a tumour‐specific marker to identify tdEVs is currently lacking. Moreover, EVs are relatively small, with sizes ranging from ∼30 nm to a few micrometres, and therefore contain low biomolecule content compared to cells.

Various strategies have been explored to isolate and detect circulating tdEVs from cancer patients. These strategies include targeting proteins commonly found on EVs, such as the tetraspanins CD9, CD63 or CD81, and proteins that are overexpressed by carcinomas, like the epithelial cellular adhesion molecule (EpCAM) (Nanou et al. 2018, 2020; Lee et al. 2023; Odaka et al. 2022). However, these markers are not exclusively cancer‐specific and are limited by the low number of epitopes on tdEVs, posing a challenge for efficient detection. Identifying a tdEV‐specific marker with an increased number of epitopes could significantly enhance detection sensitivity and provide a valuable tool for biomarker exploitation. Furthermore, such marker would enable the isolation of tdEVs, allowing for a detailed examination of their biochemical and structural composition, as well as their contribution to metastasis.

Altered glycosylation in tumours has been suggested as a hallmark of cancer (Munkley et al. 2016). As these post‐translational modifications are present on a range of different proteins, they potentially offer an abundance of novel epitopes displayed on (circulating) tdEVs. Previous studies have demonstrated the ubiquitous presence of a specific glycosaminoglycan, named oncofoetal Chondroitin Sulphate (ofCS), in malignant tissues, with limited expression in healthy tissues except the placenta (Salanti et al. 2015). OfCS can be targeted by a recombinant version of the malaria parasite protein VAR2CSA (rVAR2), or by a recently developed antibody named C9 (Vidal‐Calvo et al. 2024), with high specificity and nanomolar affinity (Salanti et al. 2015; Agerbæk et al. 2018). As a circulating cancer biomarker, cell‐free ofCS‐carrying proteoglycans has been used for pan‐cancer detection in plasma (Zhang et al. 2023) and bladder cancer detection in urine samples (Clausen et al. 2020). Furthermore, ofCS has been used to identify and isolate circulating tumor cells (CTCs) from a wide range of cancer patients, including those with glioblastoma (Bang‐Christensen et al. 2019), prostate, colorectal, and hepatic cancers (Agerbæk et al. 2018), as well as pancreatic ductal adenocarcinoma (PDAC) (Tang et al. 2025; Tang et al. 2024). Although promising, the low frequency or even absence of CTCs in routine blood draws from cancer patients has limited their clinical utility. In contrast, circulating tdEVs are suggested to be more abundant than CTCs (Nanou et al. 2018). We hypothesize that ofCS is displayed on the surface of tdEVs, thereby allowing the detection of ofCS‐modified tdEVs by rVAR2.

Here, we show that tumour‐derived EVs (tdEVs) can be identified using the rVAR2 protein coupled to a fluorophore. Using this approach, we identified tdEVs from in vitro cultured cancer cells and from plasma samples of patients with pancreatic ductal adenocarcinoma (PDAC), employing super‐resolution fluorescence microscopy (FM) and flow cytometry (FCM). This proof‐of‐concept study shows that the selective binding of rVAR2 to ofCS provides a method for identifying tdEVs, which can be used to investigate the functional roles and biomarker potential of tdEVs in cancer.

## 2 Materials and Methods

### 2.1 Cell Culture

A549 lung adenocarcinoma (ATCC: CCL‐185, RRID:CVCL_0023), PANC‐1 pancreatic cancer (ATCC: CRL‐1469, RRID:CVCL_0480), MCF‐7 breast cancer (ATCC: HTB‐22, RRID: CVCL_0031), U87mg glioblastoma (ATCC: HTB‐14, RRID: CVCL_0022), Colo205 colon cancer (ATCC: CCL‐222, RRID: CVCL_0218), ES‐2 ovarian cancer (ATCC: CRL‐1978, RRID: CVCL_3509), A375 melanoma (ATCC: CRL‐1619, RRID: CVCL_0132), HMEC primary mammary epithelial (ATCC: PCS‐600‐010), MCF10A breast epithelial (ATCC: HTB‐22, RRID: CVCL_0031) and HaCaT keratinocyte (RRID: CVCL_0038) cells were used to produce EVs.

A549, PANC‐1, MCF‐7, U87‐MG, A375 and HaCaT cells were cultured in Dulbecco's Modified Eagle Medium (DMEM, Gibco, 41966) supplemented with 10% (v/v) foetal bovine serum (FBS, BioWest, S1810). Colo205 cells were cultured in RPMI‐1640 Medium (Life Technologies, 21875‐091) and ES‐2 cells in McCoy's 5A (Modified) Medium (Life Technologies, 16600082), both supplemented with 10% FBS. HMEC cells were cultured in 0.1% gelatine‐coated flasks (Sigma, G1890) using MCDB 131 Medium (Life Technologies, 10372019), supplemented with 10 mM L‐glutamine (Life technologies, 25030024), 10 ng/mL epidermal growth factor (EGF, Corning, 354001), 1 µg/mL hydrocortisone (Sigma, H0888) and 10%–15% FBS. MCF10A cells (generously provided by F. Miller, Barbera Ann Karmanos Cancer Institute, Detroit, USA) were cultured in DMEM/Nutrient Mixture F‐12 (DMEM‐F12, Life Technologies, 31331‐028) supplemented with 5% Horse serum (Gibco, 16050‐023), 20 ng/mL EGF (Upstate Biotechnology, 01–107), 0.5 µg/mL hydrocortisone (Sigma, H‐4001), 10 µg/mL insulin (Sigma, I‐6634) and 105 ng/mL Cholera enterotoxin (Calbiochem, 227035). Additionally, all cell culture media were supplemented with 0.2% penicillin‐streptomycin (pen/strep) (Thermo Fisher Scientific, 15140163), and cells were maintained at 37°C in a humidified atmosphere with 5% CO_2_. Cells were seeded in T175 flasks (Cellstar Grenier, 660175) at an initial cell density within the range recommended by ATCC. The medium was refreshed twice per week. All cell lines were tested for the absence of mycoplasma.

### 2.2 EV Collection

At 70% confluence, cells were washed three times with 1× phosphate‐buffered solution (PBS) and cultured in DMEM supplemented with 0.2% pen/strep, and either depleted of FBS (A549 cells), or supplemented with 10% filtered FBS (PANC‐1) that was filtered using AmiconUltra‐15 (cut‐off: 100 kDa). After 48 h of incubation at 37°C and 5% CO_2_, the conditioned medium was collected in 50 mL tubes (Cellstar Grenier, 227261). To remove detached cells and cell debris, the conditioned medium was first centrifuged at 300 × *g* for 5 min at 4°C. Next, the supernatant was collected and centrifuged at 2000 × *g* for 15 min at 4°C. Ultrafiltration was performed using tangential flow filtration cartridges (TFF‐Easy filtration cartridge, HansaBioMed, HBM‐TFF/1) to increase the concentration of EVs in the conditioned medium. This gentle method avoids aggregation of particles and soluble proteins. EVs were concentrated either manually using syringes or by a peristaltic pump (Masterflex) at a flow rate of 20 mL/min and tubing size 13 (Masterflex). Next, the EV‐containing medium buffer was exchanged to Dulbecco's Phosphate Buffered Saline (DPBS, Corning Dulbecco's Phosphate‐Buffered Saline, #21‐031‐CV) with 3% lipoprotein‐depleted FBS (filtered using AmiconUltra‐15, cut‐off: 100 kDa, Cat. No.: UFC9100) until a final volume of 1–3 mL was reached. Samples were then distributed in aliquots, snap‐frozen in liquid N_2_ and stored at −80°C until use.

### 2.3 EV Sample Labelling

#### 2.3.1 Protein Production and Fluorophore Labelling of rVAR2

The recombinant truncated rVAR2CSA protein (rVAR2) consisting of the DBL1‐ID2a domains, C‐terminal 6x‐histidine and V5‐tags as well as an N‐terminal SpyTag was expressed in Shuffle T7 Express Competent *E. coli* (NEB). The protein was purified using two chromatography steps, first an affinity step on HisTrap HP (Cytiva) and subsequently a cation exchange step on HiTrap SP HP (Cytiva) as previously described (Salanti et al. 2015). The purity of the produced protein was analysed by sodium dodecyl‐sulphate polyacrylamide gel electrophoresis (SDS‐PAGE), showing a single band at 121 kDa. The rVAR2 specificity for ofCS was validated by binding to decorin using ELISA, as well as binding to cancer cells using flow cytometry as previously described (Sand et al. 2020).

The SpyCatcher protein with a C‐terminal 6x‐histidine tag was produced in *E. coli* BL21 DE3 (NEB, C2527H) and purified using two chromatography steps, first an affinity step on HisTrap HP (Cytiva) and subsequently an anion exchange step on HiTrap Q HP(Cytiva). The purified SpyCatcher protein was labelled with a fluorophore using the Alexa Fluor 647 NHS Ester dye kit (Invitrogen, A20006) according to the protocol of the manufacturer. Effective Alexa647‐Spycatcher binding to rVAR2 was evaluated and confirmed by SDS page (Figure S1).

The rVAR2 protein and the SpyCatcher‐Alexa 647 were mixed at a 1:1.2 molar ratio and incubated for 1 h at room temperature (RT) protected from light to generate rVAR2‐AF647. This conjugation utilizes the SpyTag‐SpyCatcher technology (Zakeri et al. 2012) and ensures that all rVAR2 has an Alexa647 conjugated SpyCatcher. Successful conjugation was confirmed with SDS‐PAGE.

Chondroitinase ABC (chABC, Uniprot P59807, RRID:SCR_002380) was used to digest ofCS in negative control samples (Figure S2C). This enzyme with a C‐terminal 6x‐histidine tag was expressed in *E. coli* Shuffle T7 Express cells (NEB, C3029J). chABC was purified using two chromatography steps, first an affinity step on HisTrap HP (Cytiva) and subsequently size exclusion using a HiPrep 16/60 Sephacryl S‐300 HR column (Cytiva).

#### 2.3.2 Labelling

Reagents (rVAR2‐AF647 and anti‐CD81‐PE/‐AF488, Clone M38, Invitrogen, Cat. No.: A15781, RRID:AB_2534560 (PE), MA5‐44132, RRID:AB_2913064 (AF488)) were centrifuged at 19,000 × *g* for 5 min to eliminate protein aggregates. After centrifugation, the supernatants were carefully collected, leaving 10–15 µL of reagents in the tube. Next, rVAR2‐AF647 was added to the samples at a final concentration of 800 nM, followed by the addition of NaCl at a final concentration of 150 mM. Anti‐CD81‐PE/‐AF488 was added to the samples at a final concentration of 2 µg/mL to facilitate the visualisation of EVs during fluorescence microscopy. Samples were incubated for 2 h in the dark at RT. Thereafter, the samples were kept on ice in the dark until use. The workflow is shown in Figure S2B.

To confirm tdEV detection in plasma, A549‐derived EVs were spiked directly into plasma collected from healthy donors and thereafter labelled with both rVAR2‐AF647 and anti‐CD81‐PE antibody, as previously described.

#### 2.3.3 Chondroitinase Controls

Prior to labelling, the EV samples were treated with 50 µg/mL chABC or vehicle control (DPBS) for 1 h at RT, whereas the cancer cells were incubated for 30 min at 37°C in 20 µg/mL chABC diluted in DPBS + 2% FBS (Gibco).

#### 2.3.4 EV Capture on Functionalised Surfaces

To validate the rVAR2 binding to ofCS on tdEVs, EVs were captured and labelled on microfluidic chips (EV profiler 2, ONI, UK) (Moon et al. 2024; Wolf et al. 2022) with bottom surfaces that were coated with a tetraspanin trio (TT) targeting CD9, CD63 and CD81. Sample processing was performed in accordance with the instructions of the manufacturer. In brief, a multilane chip was functionalized with the TT followed by a washing step. A549‐derived EVs and negative control samples (EVs pre‐treated with chABC and procedural controls) were then loaded to the chip (10 µL per lane). The chip was incubated for 75 min at RT, while rocking at 30–45 RPM in a direction parallel to the lanes. After incubation, all lanes were washed, fixed for 10 min, and then washed again. Next, 10 µL of a mix of 800 nM rVAR2‐AF647, 150 mM NaCl and 2 µg/mL CD81‐AF488 were added to all lanes. After an incubation time of 50 min, all lanes were washed. During all incubation steps, the chip was kept in a humidity chamber. The buffer in the lanes was exchanged with the dSTORM buffer (ONI, UK), prior to imaging with direct stochastic optical reconstruction microscopy (dSTORM). Workflow is shown in Figure S2A.

#### 2.3.5 Size Exclusion Chromatography

Samples analysed with Airyscan fluorescence microscpy (FM) and flow cytometry (FCM) were labelled directly in suspension, followed by size exclusion chromatography (SEC) to isolate EV‐rich fractions and remove unbound reagents. The Izon fraction collector was used with 35‐nm SEC columns (qEVoriginal Gen 2, Izon Science, New Zealand). A maximum of 500 µL of labelled sample was loaded into the column, followed by Dulbecco's phosphate buffered saline (DPBS) as a flushing buffer. After a flow‐through of 2.9 mL, 400 µL EV fractions were collected, and the second fraction containing the highest concentration of EVs was analysed. For patient 1 sample and respective healthy control sample, pooled fractions 2–4 were analysed due to a technical issue with the SEC fraction collector. The fractions were stored at 4°C in the dark. The workflow is shown in Figure S2B.

### 2.4 Sample Processing of EVs Derived From TGF‐β‐Treated A549 Cells

To assess whether ofCS on tdEVs is conserved during epithelial to mesenchymal transition (EMT), epithelial A549 cancer cells were stimulated with recombinant human transforming growth factor‐β3 (TGF‐β) to induce EMT (Fan et al. 2023). To confirm EMT induction in A549 cells following TGF‐β stimulation, we evaluated changes in cell morphology along with epithelial and mesenchymal marker expression.

#### 2.4.1 F‐Actin Fluorescent Staining

Fluorescence staining was conducted to assess the expression and localisation of F‐actin using a previously established protocol (Fan et al. 2023). In brief, A549 cells were stimulated with TGF‐β at a concentration of 1 ng/mL, or with the corresponding vehicle for 48 h. The fixed cells were then stained with phalloidin conjugated to Alexa Fluor 488 (1:500 dilution; Thermo Fisher Scientific, A12379) for 30 min at RT. VECTASHIELD Antifade Mounting Medium containing DAPI (4′,6‐diamidino‐2‐phenylindole; Vector Laboratories, H‐1200) was used for mounting the coverslips. Images were acquired using a Leica SP8 confocal microscope (Leica Microsystems).

#### 2.4.2 Western Blotting

Cells were lysed using Radioimmunoprecipitation assay buffer (RIPA) buffer, which contains 150 mM sodium chloride, 1.0% Triton X‐100, 0.5% sodium deoxycholate, 0.1% SDS, and 50 mM Tris‐HCl (pH 8.0). A complete protease inhibitor cocktail (Roche; Cat. No.: 11836153001) was included in the buffer. Protein concentrations were measured using the DC protein assay kit (Bio‐Rad; Cat. No.: 5000111) following the manufacturer's instructions. For separation, equal amounts of protein were loaded onto SDS‐PAGE. After electrophoresis, proteins were transferred to a 0.45‐µm polyvinylidene difluoride membrane (Merck Millipore; Cat. No.: IPVH00010). The membrane was blocked for 1 h at RT using a 5% non‐fat dry milk solution dissolved in Tris‐buffered saline (TBS) with 0.1% Tween 20 (TBST). Following blocking, the membranes were probed with the appropriate primary and secondary antibodies. Signal detection was achieved using Clarity Western ECL Substrate (Bio‐Rad; Cat. No.: 1705060) and a ChemiDoc Imaging System (Bio‐Rad; Cat. No.: 17001402). Recombinant TGF‐β3 was a gift from Dr. A. Hinck (University of Pittsburgh, USA).

#### 2.4.3 EV Collection From TGF‐β‐Induced EMT of A549 Cells

To induce EMT, adherent A549 cells were washed twice with PBS at 70% confluence and cultured in FBS‐free DMEM containing TGF‐β (2.5 ng/mL) or vehicle control. After 48 h, the conditioned medium was collected. Cells were removed and EVs were concentrated using AmiconUltra‐15 (cut‐off: 100 kDa) and labelled, as previously described. A chABC control at 331 µg/mL was included.

### 2.5 Collection and Processing of Plasma From PDAC Patients and Healthy Donors

Peripheral blood samples were obtained from a cohort of five patients with stage III or IV PDAC (Table ST1). Samples were collected with written informed consent and approved by the medical‐ethical assessment committee of the Academic Medical Center, University of Amsterdam (METC number: 2013_315). None of the patients included had undergone chemo‐ or radio‐therapy at the time of inclusion and blood collection.

Peripheral blood of healthy donors was obtained via the Leiden University Medical Center healthy voluntary donor service (LuVDS) and Academic Medical Center, University of Amsterdam (Table ST2). All healthy donors gave broad consent. The biomaterial and associated clinical data of all healthy donors collected in the LuVDS are released for research purposes only, after being approved by the internal review board. Blood samples of healthy donors from the Academic Medical Center were collected in accordance with the guidelines of the Medical Ethical Committee of the Academic Medical Center, University of Amsterdam (W22‐243 #22.298).

Peripheral blood samples were collected from both patients and healthy donors using Ethylenediaminetetraacetic acid (EDTA) and/or Citrate tubes (Vacutainer, Becton Dickinson, USA). Whole blood from healthy donors was centrifuged twice at 2500 × *g* for 15 min at 20°C, and the plasma was pooled (Coumans et al. 2017). Plasma obtained at the Leiden University Medical Center was filtered using an 800 nm filter (Isopore, ATTP02500, Merck Milipore, Germany). Similar to healthy plasma, biobank patient plasma was previously prepared with a double centrifugation protocol at 1550 × *g* for 20 min at 20°C. All samples were centrifuged without break, and the plasma fractions were carefully collected from approximately 10 mm above the buffy coat or cell pellet to minimize platelet contamination. Plasma samples were distributed in aliquots with a volume of <500 µL, snap‐frozen in liquid N_2_ and stored at –80°C until use.

### 2.6 Sample Analysis and Data Processing

#### 2.6.1 Super‐Resolution dSTORM Fluorescence Microscopy Imaging

A549 EVs captured on the EV profiler chip were imaged with dSTORM, achieving a lateral resolution of 20 nm (Moon et al. 2024; Wolf et al. 2022). Images were acquired in a Nanoimager (ONI, Oxford, UK) using NimOS Nanoimager Software (ONI, UK). A 100 × 1.4 NA oil immersion objective (Olympus) was used for emission and collection of the laser light. During image acquisition, 40% of the laser power was used for both 488 and 640 nm lasers. Seven thousand frames were acquired with an exposure time of 30 ms per frame. Images were analysed (*n* = 3) using the EV profiling essentials settings in the CODI cloud platform (https://alto.codi.bio/, ONI, UK). In addition, a maximum radius of 90 nm around the centroid and a minimum of four localisations per channel determined positivity.

#### 2.6.2 Super‐Resolution Airyscan Fluorescence Microscopy (FM) Imaging

EV‐containing SEC fractions (3–4 µL) were placed on a coverslip (VWR, 631‐0153), which was loaded on a glass slide (Avantor, VWR Microscope slides) and imaged with a microscope (LSM 900 Airyscan 2, Zeiss, Germany) using a Plan‐Apochromat 63×/1.4 oil immersion objective and the super‐resolution Airyscan mode. Images were acquired using 488, 561 and 640 nm lasers at 3.7%, 5.0% and 4.0% power, respectively, a scan speed of 5, and a detector gain of 800 V. The image size was 78.01 × 78.01 µm with a pixel size of 0.43 µm. Once the samples were in focus, images were taken at predefined locations in the slide to prevent operator image selection (Rikkert et al. 2019). To confirm EV detection, we treated the samples with 1% Nonidet P‐40 (NP40). As EVs have a phospholipid membrane that is sensitive to detergent, NP40 (partially) lyses EVs (Figure S2D) and causes a reduction in fluorescence intensity, whereas detergent‐insoluble particles like proteins remain intact (Welsh et al. 2024). Fluorescence images were analysed with the Zeiss Zen Blue software (v3.8). Furthermore, frequency histograms of the rVAR2‐AF647 and CD81‐AF488 signal intensities of all the pixels were extracted from each image using a bin size of two. A threshold of 40 a.u. was applied to account for background, and the areas under the histograms were computed for at least three representative images per sample. FM bar plots in this paper show the areas under the histograms representing the rVAR2 mean fluorescence intensity per image. Data are presented as mean with standard deviation error bars. Images with visible artefacts, such as out‐of‐focus areas or glass defects, were excluded from the analysis.

#### 2.6.3 Flow Cytometry (FCM) Measurements

FCM measurements were performed using a flow cytometer (Northern Lights, Cytek Biosciences, USA). EV‐containing SEC fractions were diluted in DPBS to ensure an event rate below 30,000 counts/s and avoid swarm detection (Buntsma et al. 2023). EV samples from cancer cell lines were measured for 120 s, while plasma samples were measured for 360 s. The trigger was set on the R2‐fluorescence detector, which detects the rVAR2‐AF647 signal between 669 and 687 nm with 640‐nm illumination. To determine the fluorescence threshold, unstained EV samples were measured with the trigger set on the side scattering (SSC) detector using a threshold of 1500 arbitrary units (a.u.), corresponding to a side scattering cross section of ∼2 nm^2^. The R2‐fluorescence threshold was then set to 1000 a.u. (∼165 molecules of equivalent soluble fluorochrome (MESF)), corresponding to the upper R2‐fluorescence boundary of unstained EV samples and healthy plasma. To assess the stability of the background noise of R2‐fluorescence, an unstained sample was measured each time alongside the labelled samples.

Fluorescence calibration was performed to relate arbitrary units of fluorescence to standard units of MESF (Welsh et al. 2023). For this, Rainbow calibration particles (SPHERO, lot EAP01, Spherotech Inc) were cross‐calibrated against MESF beads conjugated to AF647 and measured daily. Further details are reported in the supplemented MIFlowCyt‐EV. Following light scattering and fluorescence calibration, FCM data were analysed using Falcon (v0.56, Exometry, The Netherlands) and FlowJo^TM^ v10.9 Software (BD Life Sciences). Counts were related to concentrations by accounting for sample dilution and the analysed volume, determined using a calibrated flow rate sensor. Fluorescence intensity histograms were expressed in MESF units. To quantify the rVAR2 signal, we computed the area under the histogram, representing the total rVAR2‐associated fluorescence per unit volume (MESF/mL). FCM bar plots in this study show the total rVAR2‐associated signal in MESF per millilitre of fluid.

### 2.7 Statistical Analysis

Statistical analysis and plots were generated in GraphPad Prism (v9.3.1, USA). All FM images were analysed using one‐way Analysis of Variance (ANOVA) followed by Tukey's HSD multiple comparisons, with *p < 0.05 considered statistically significant. A corresponding healthy sample was analysed on the same day for each patient sample. Patient‐to‐healthy area ratios were then determined for each patient‐healthy pair, log‐transformed and analysed using a one‐sample *t*‐test.

## 3 Results

### 3.1 rVAR2 Binds to ofCS Displayed on A549 Lung Cancer Cells and Their EVs

**FIGURE 1:**
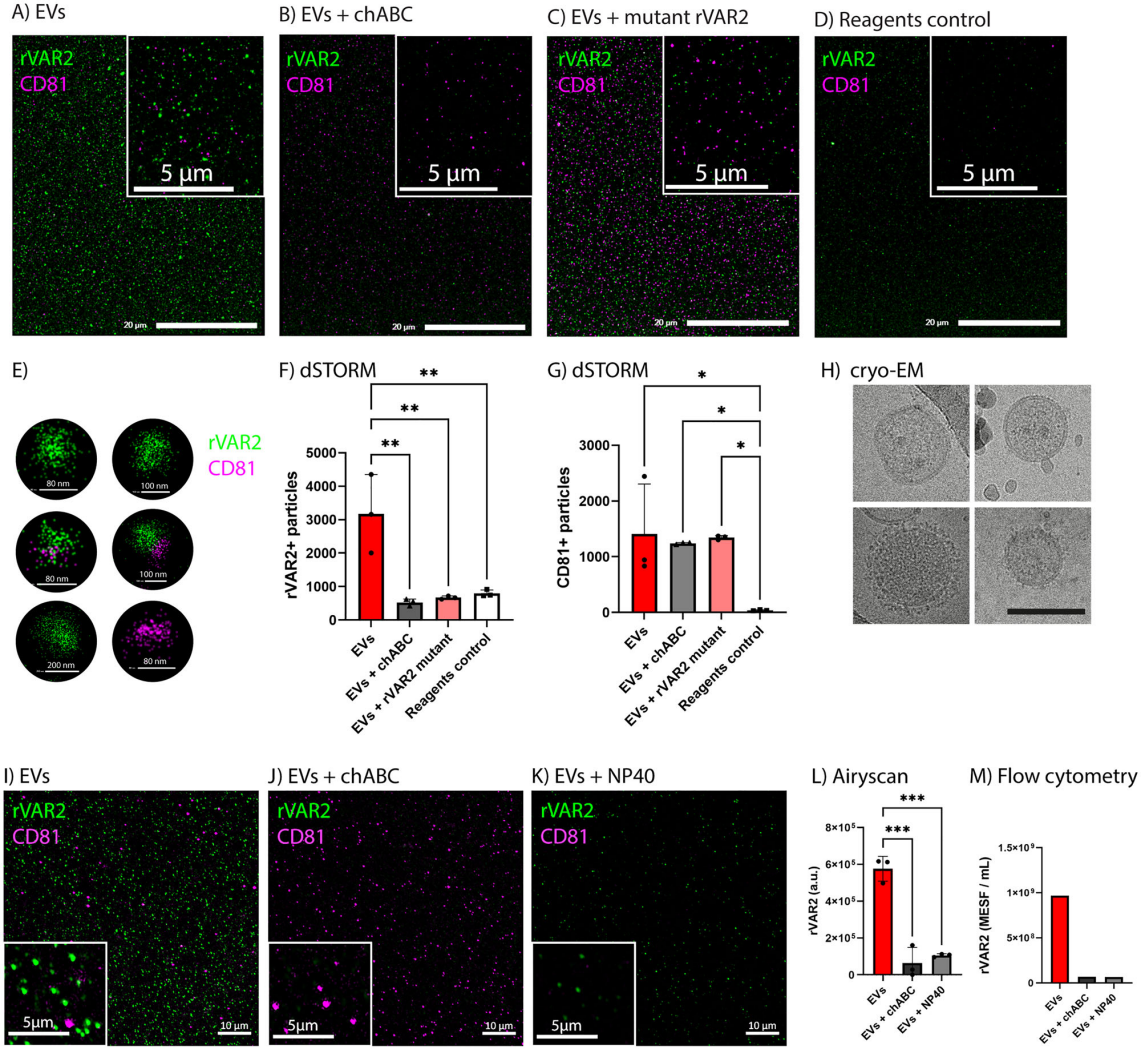
rVAR2 binds to ofCS present on lung cancer A549‐derived EVs. (A–C) A549‐derived EVs captured using anti‐CD9, anti‐CD63 and anti‐CD81 antibodies, and detected through dSTORM. (A) A549‐derived EVs labelled with rVAR2. (B) chABC‐treated A549‐derived EVs labelled with rVAR2. (C) A549‐derived EVs labelled with rVAR2 mutant. (D) Procedural control (no EVs, only reagents in buffer). Scale bars in the main images (A–D) and insets represent 20 µm and 5 µm, respectively. (E) dSTORM images of single EVs stained with rVAR2 and CD81 antibody. Individual EVs were identified using the CODI platform clustering analysis (EV profiling essentials), which groups single‐molecule localisations into distinct EVs. Due to the nature of super‐resolution single‐molecule localisation microscopy, each EV appears as a cluster of individual localisations rather than a continuous structure. rVAR2 is shown in green and CD81 in magenta for all FM images. CD81 labelling was included to aid the visualisation of chABC controls. (F, G) Average number of single (F) rVAR2^+^ or (G) CD81^+^ particles counted per image across different conditions (*n* = 3 images per condition). (H) Representative cryo‐electron microscopy images of A549‐derived EVs after isolation following the workflow in Figure S2B. Scale bar represents 100 nm. (I) A549‐derived EVs labelled with rVAR2, (J) EVs treated with chABC before labelling and (K) EVs treated with NP40 after labelling, and detected through Airyscan fluorescence microscopy. Scale bars in the main images and insets represent 10 µm and 5 µm, respectively. (L) rVAR2 mean fluorescence intensity of three representative images per condition. Statistical analysis was performed using one‐way ANOVA with Tukey's multiple comparisons test (* = *p* < 0.05, ** = *p* < 0.01, *** = *p* < 0.001). For all microscopy bar plots, error bars represent standard deviation. (M) rVAR2‐associated fluorescence signal, measured by FCM and expressed in MESF per millilitre, for A549‐derived EV samples with corresponding chABC and NP40 controls.

Cancer cells express oncofoetal Chondroitin Sulphate (ofCS), which can be specifically targeted by rVAR2 (Salanti et al. 2015, Agerbæk et al. 2018). We hypothesised that tdEVs expose ofCS. To evaluate the potential of using rVAR2 for tdEV detection, we used the A549 lung cancer cell line as a source of tdEVs. We first confirmed the specific binding of rVAR2 to the A549 cell surface (Figure S3A‐C). Next, tdEVs were captured on a surface coated with antibodies against tetraspanins, followed by rVAR2 labelling (Figure 1A,E). To test the labelling specificity of rVAR2, we used a chABC enzyme that selectively digests chondroitin sulphate (CS, Figure S2C). Treatment of tdEVs with chABC prior to rVAR2 and anti‐CD81 labelling nearly abolished the binding of rVAR2 but not of anti‐CD81 (Figure 1B,F,G). Similar results were obtained in a phosphatidyl serine (PS)‐based captured method (Figure S3G‐J). To further validate the binding specificity of rVAR2 to ofCS and exclude potential non‐specific interactions, we labelled tdEVs with a mutated version of rVAR2 (Wang et al. 2021) (rVAR2 mutant), which has a decreased binding to ofCS. We observed a significant decrease in the number of tdEVs labelled with the rVAR2 mutant compared to tdEVs labelled with rVAR2, with no significant difference in the number of anti‐CD81^+^ tdEVs (Figure 1C,F,G). These results indicate that the binding of rVAR2 to the tdEVs is CS‐dependent, and not a result of unspecific protein binding. A procedural control, in which only reagents and no tdEVs were added to the chip, showed that the number of fluorescent particles identified was significantly lower than in the rVAR2^+^ EV sample (Figure 1D,F,G). This indicates that the reagents contribute minimally to the fluorescence signals. In addition, high‐throughput cryo‐electron microscopy (Supporting Information Methods) confirmed the presence of A549‐derived EVs and demonstrated good sample quality (Figure 1H). While some darker particles are visible in cryo‐EM images (examples are indicated by the red arrows in Figure S4A), these are consistent with ice artefacts, which are commonly formed during sample preparation for cryo‐EM analysis. To rule out lipoprotein contamination, 139 cryo‐EM images of our EV preparations were inspected, and did not observe any detectable lipoprotein contamination (Figure S4A‐C).

Next, we evaluated the rVAR2 binding to tdEVs directly in suspension using an orthogonal method, namely Airyscan super‐resolution FM. Consistent with our previous results, Figure 1I,L show the binding of rVAR2 to tdEVs, whereas the treatment of tdEVs with chABC nearly abolished the rVAR2 signal (Figure 1J,L), but left CD81 labelling unaffected (Figure 1J). To further validate that the labelled particles were lipid‐based vesicles and not protein aggregates, we applied the detergent treatment control with a nonionic, non‐denaturing detergent (NP40). This treatment resulted in the (partial) lysis of the EV membranes, leading to a reduction in the number of rVAR2^+^ and anti‐CD81^+^ particles along with an observed decrease in fluorescence signal (Figure 1K,L). As the rVAR2 signal was not fully abolished when adding the detergent, we tested whether NP40 interfered with the binding of rVAR2 to ofCS in an enzyme‐linked immunosorbent assay (ELISA) (Supporting Information Methods). As illustrated in Figure S5, the binding of rVAR2 to CS‐carrying decorin was not inhibited in the presence of NP40 (Supporting Information Methods), suggesting that the decrease in both rVAR2^+^ particles and the fluorescence signal resulted from EV (partial) lysis. Procedural controls where DPBS with lipoprotein‐depleted FBS was stained for rVAR2 and CD81 show a virtual absence of fluorescence signals (Figure S4D,E), confirming that our protocol ensures specific detection with minimal background due to reagents and buffers.

To further validate our findings, we assessed the rVAR2 binding to ofCS‐displaying tdEVs using FCM. Figure 1M shows the rVAR2‐associated fluorescence signal expressed in MESF per millilitre of sample containing A549‐derived EVs. These results confirm that rVAR2 binds to tdEVs, which are susceptible to lysis during NP40 treatment. Furthermore, rVAR2 did not appear to have distinct binding to CD81^+^ or CD63^+^ EVs (Figure S6).

### 3.2 ofCS Is Retained on A549‐Derived EVs Following Epithelial‐to‐Mesenchymal Transition

**FIGURE 2:**
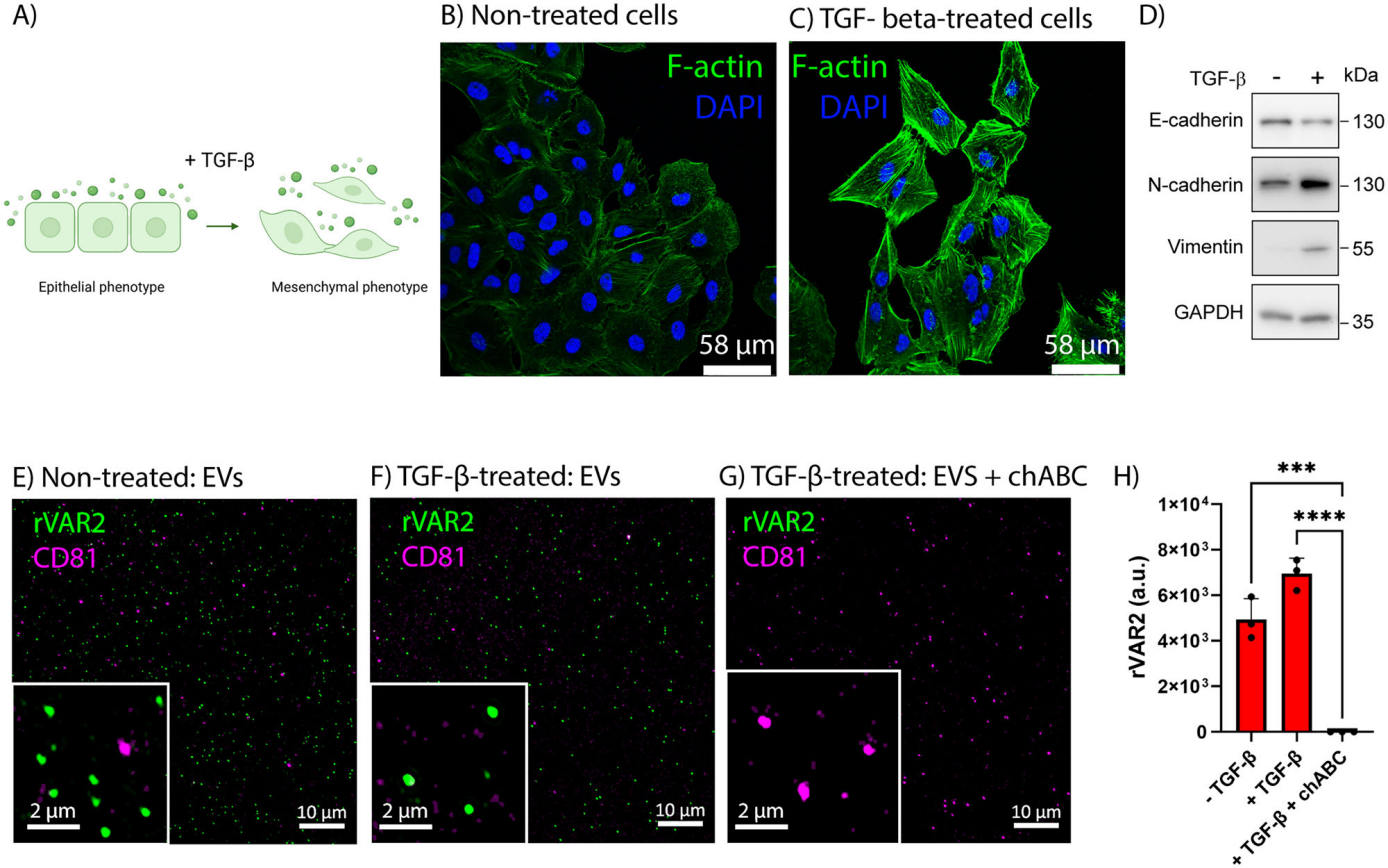
OfCS is retained on tdEVs following epithelial‐to‐mesenchymal transition of A549 lung adenocarcinoma cells. (A) Schematic representation of epithelial‐mesenchymal transition (EMT) induction by TGF‐β stimulation of cells. Epithelial cells (left) show a cobblestone‐like morphology with strong cell‐cell adhesion, while mesenchymal cells (right) appear elongated and spindle‐shaped, with enhanced migratory properties. (B and C) Fluorescence microscopy images of A549 cells stained for F‐actin and DAPI before (B) and after (C) TGF‐β treatment. (D) Western blot analysis of A549 cells before and after TGF‐β stimulation showing the downregulation of epithelial markers (E‐cadherin) and the upregulation of mesenchymal markers (N‐cadherin, Vimentin). (E) rVAR2 labelling of EVs derived from vehicle‐treated A549 cells. (F) rVAR2 labelling of EVs derived from TGF‐β‐treated A549 cells. (G) chABC control for EVs derived from TGF‐β‐treated A549 cells. Scale bars in the main images and insets represent 10 µm and 2 µm, respectively. (H) rVAR2 mean fluorescence intensity of three representative images per condition. Statistical analysis was performed using one‐way ANOVA with Tukey's multiple comparisons test (* = *p *< 0.05, ** = *p *< 0.01, *** = *p *< 0.001, **** = *p *< 0.0001). Error bars represent standard deviation.

Cancer cells change their phenotype during epithelial‐to‐mesenchymal transition (EMT, Figure 2A), to facilitate metastasis and therapy resistance. The retention of ofCS on tdEVs even after EMT ensures that tdEV detection is not limited to EVs from epithelial‐like tumour cells, thereby broadening their clinical applicability. To assess whether ofCS remains detectable on tdEVs from mesenchymal‐like cells, we collected tdEVs from A549 cells pre‐treated with TGF‐β to stimulate EMT. To confirm that TGF‐β stimulation induced EMT in A549 cells, we assessed cell morphology, as well as epithelial and mesenchymal marker expression. Figure 2B,C revealed a clear morphological shift, with cells becoming elongated and spindle‐shaped, consistent with EMT. As expected, TGF‐β treatment led to the downregulation of epithelial markers such as E‐cadherin and the upregulation of mesenchymal markers including N‐cadherin and Vimentin (Figure 2D). Despite this transition, ofCS remained present on tdEVs from both untreated and TGF‐β‐treated cells. As shown in Figure 2E–H, rVAR2 labelled tdEVs from both non‐treated and TGF‐β‐treated cells, whereas the rVAR2 signal was abolished in the chABC control (Figure 2G,H).

### 3.3 rVAR2 Binds to ofCS Displayed on tdEVs From Diverse Cancer Cell Lines

**FIGURE 3:**
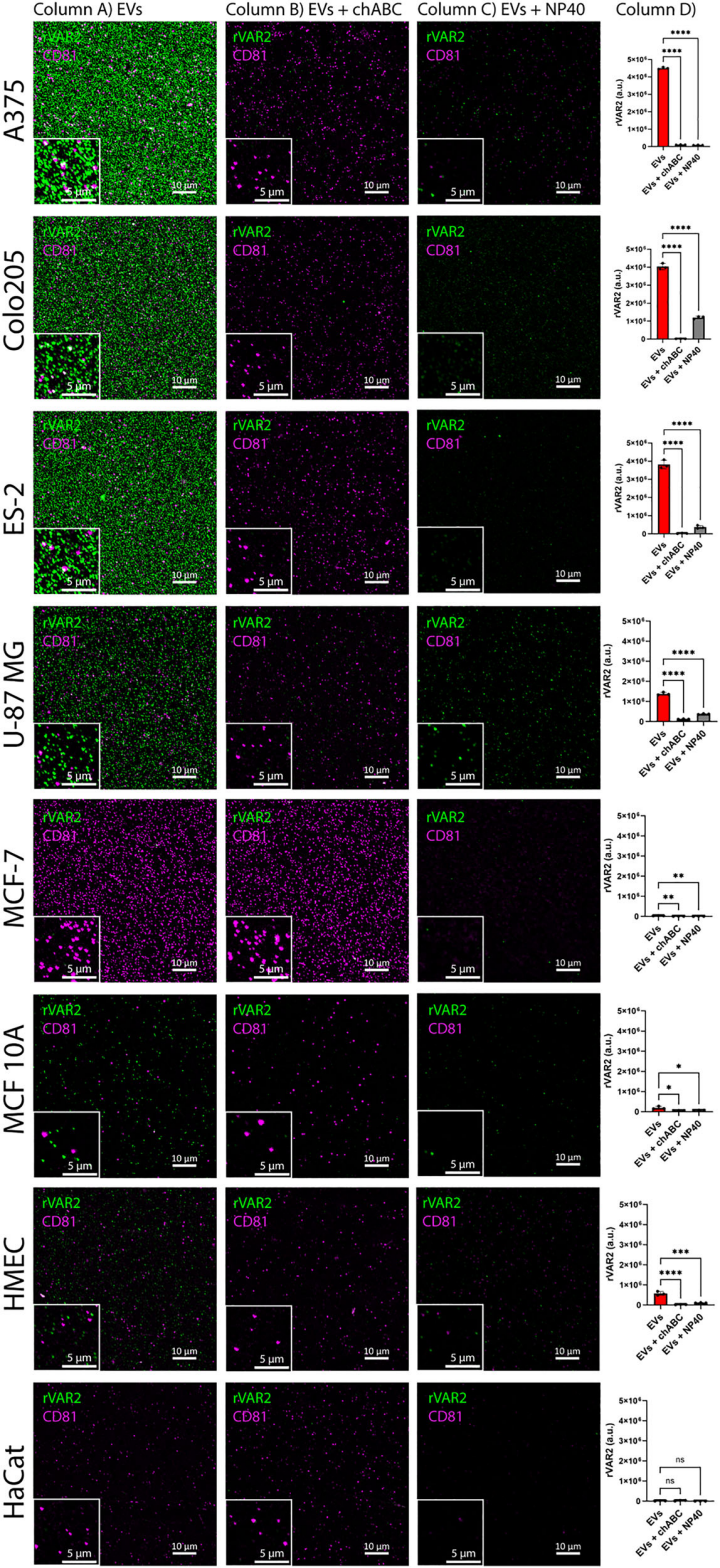
rVAR2 labelling of EVs from various malignant and non‐malignant cell lines. Airyscan fluorescence microcopy images at different conditions: (Column A) EVs labelled with rVAR2, (Column B) EVs treated with chABC before labelling, and (Column C) EVs treated with NP40 after labelling. Rows represent different cell lines, that is, melanoma (A375), colorectal cancer (Colo205), ovarian cancer (ES‐2), glioblastoma (U87‐mg), breast cancer (MCF‐7), breast epithelial cells (MCF10A), primary mammary epithelial (HMEC) and human keratinocyte line (HaCat).Scale bars in the main images and insets represent 10 µm and 5 µm, respectively. (Column D) rVAR2 mean fluorescence intensity of three representative images per condition. Statistical analysis was performed using one‐way ANOVA with Tukey's multiple comparisons test (* = *p* < 0.05, ** = *p* < 0.01, *** = *p* < 0.001, **** = *p* < 0.0001). Error bars represent standard deviation.

We further assessed the presence of ofCS across tdEVs from some of the most common tumour types, including melanoma (A375), colorectal cancer (Colo205), ovarian cancer (ES‐2), glioblastoma (U87‐mg) and breast cancer (MCF‐7). rVAR2 labelling was detected across multiple tdEVs (Figure 3, Column A), with the highest signal observed in EVs from highly aggressive and metastatic cancer cells, that is, A375, Colo205, ES‐2 and U‐87 MG, whereas EVs from (non‐metastatic) MCF‐7 cells did not show detectable rVAR2 staining. EVs from non‐malignant cell lines, such as MCF10A and HaCaT, showed generally lower rVAR2 staining. We observed low‐level rVAR2 binding to some non‐malignant EVs, particularly those derived from HMEC cells.

The specificity of rVAR2 labelling was confirmed by enzymatic treatment with chABC, which abolished the fluorescence signal, as well as detergent lysis with NP‐40, further verifying that the detected signal originated from intact vesicles (Figure 3, Columns B and C). Quantification of fluorescence intensity (Figure 3, Column D) showed a significant reduction in rVAR2 labelling upon enzymatic or detergent treatment. These findings suggest that rVAR2 binding epitope (ofCS) is widely displayed on tdEVs from different cancer cell types. The presence of ofCS on tdEVs was further validated using C9 antibody fragments against ofCS (Supporting Information Methods), (Vidal‐Calvo et al. 2024) as shown in Figure S7 for ES‐2‐ and Colo205‐derived EVs.

### 3.4 rVAR2 Binds A549‐Derived EVs Spiked Into Plasma

**FIGURE 4:**
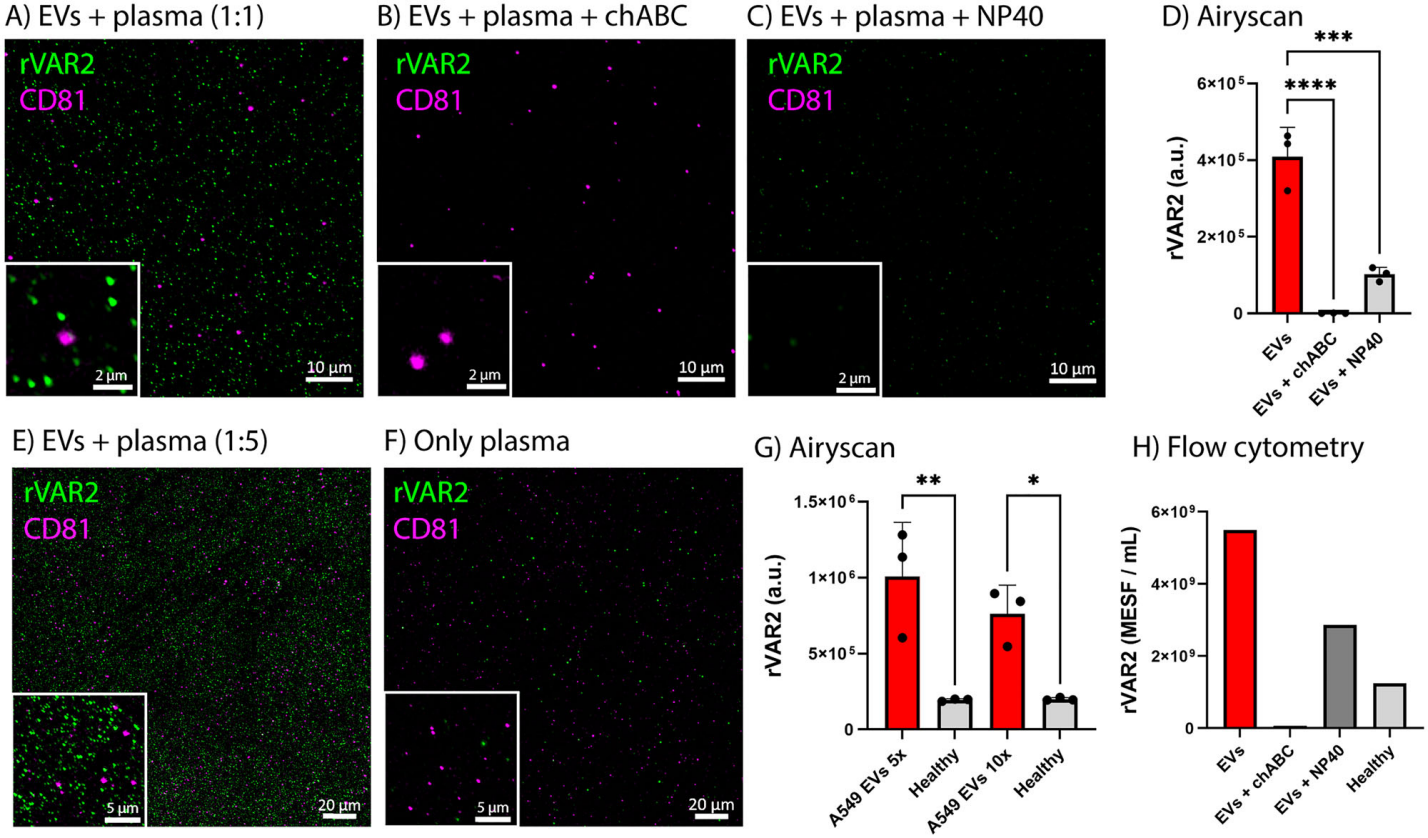
rVAR2 binds lung cancer A549‐derived EVs when spiked into plasma, as evaluated by Airyscan fluorescence microscopy (FM) and flow cytometry (FCM). (A) rVAR2 labelling of A549‐derived EVs spiked into plasma. (B) rVAR2 labelling of chABC‐treated A549‐derived EVs spiked into plasma. (C) rVAR2 labelling of A549‐derived EVs spiked in plasma, followed by treatment with NP40. Scale bars in the main images and insets represent 10 µm and 2 µm, respectively. (D) rVAR2 mean fluorescence intensity of three representative images per condition (A–C) for A549‐derived EVs spiked into plasma, with corresponding chABC and NP40 controls. (E) rVAR2 labelling of A549‐derived EVs spiked into plasma in a 1 to 5 ratio. (F) rVAR2 labelling of plasma from a healthy donor. Scale bars in the main images and insets represent 20 µm and 5 µm, respectively.  (G) rVAR2 mean fluorescence intensity of three representative images per condition. Statistical analysis was performed using one‐way ANOVA with Tukey's multiple comparisons test (* = *p* < 0.05, ** = *p* < 0.01, *** = *p* < 0.001, **** = *p* < 0.0001). Error bars represent standard deviation. (H) rVAR2‐associated fluorescence signal, measured by FCM and expressed in MESF per millilitre, for A549‐derived EVs spiked into plasma, with corresponding chABC, NP40 and healthy controls.

Given the presence of ofCS on EVs isolated from cancer cell lines, we next assessed the rVAR2‐based detection of tdEVs spiked into healthy donor plasma. Figure 4A,D show the rVAR2 labelling of A549‐derived EVs after spiking into plasma, whereas chABC treatment nearly abolished the rVAR2 signal (Figure 4B,D). Treatment of the plasma‐A549 EV mixture with NP40 reduced both rVAR2^+^ and CD81^+^ EVs, although the rVAR2 signal was not entirely removed (Figure 4C,D). We also spiked A549‐derived EVs into plasma at different dilution ratios, and successfully detected the rVAR2 signal (Figure 4E,G). In contrast, unspiked plasma samples incubated with rVAR2 showed significantly lower rVAR2 signal (Figure 4F,G). We further validated these findings using FCM. Figure 4H shows the rVAR2‐associated fluorescence signal, expressed in MESF per millilitre, in plasma with and without spiked A549‐derived EVs.

### 3.5 PANC‐1 Pancreatic Cancer Cells and Their tdEVs Exhibit Moderate ofCS Expression

**FIGURE 5:**
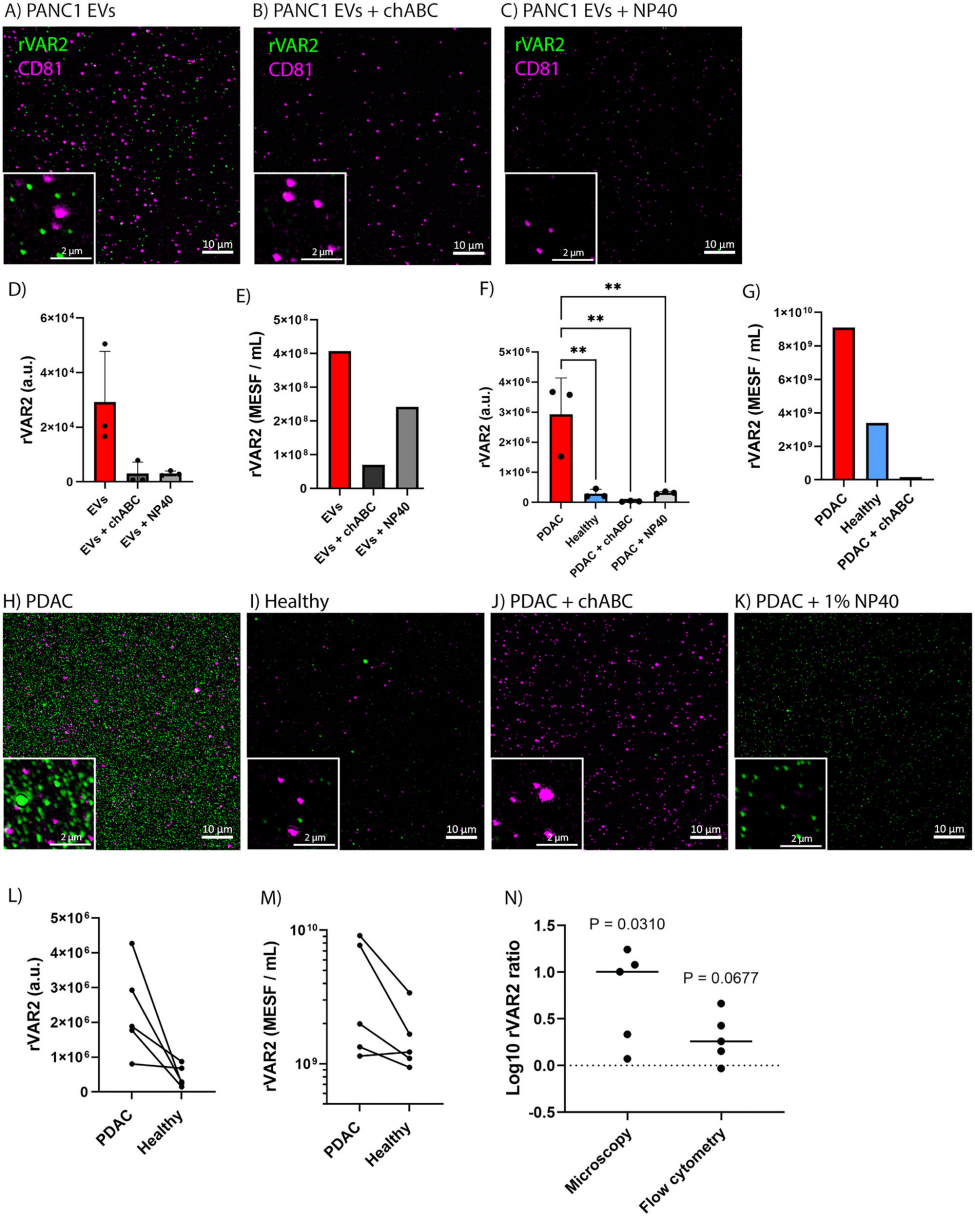
rVAR2 binds pancreatic cancer PANC‐1‐derived EVs and circulating EVs in patient plasma. (A) PANC‐1‐derived EVs labelled with rVAR2, (B) treated with chABC before labelling, and (C) treated with NP40 after labelling. Scale bars in the main images and insets represent 10 µm and 2 µm, respectively. (D) rVAR2 mean fluorescence intensity of three representative images per condition. (E) rVAR2‐associated fluorescence signal, measured by FCM and expressed in MESF per millilitre, for PANC‐1‐derived EV sampleswith corresponding chABC and NP40 controls. (F) rVAR2 mean fluorescence intensity of plasma from one PDAC patient and pooled plasma from four healthy donors labelled with rVAR2, with corresponding chABC and NP40 controls (*n* = 3 representative images per condition). Statistical analysis was performed using one‐way ANOVA with Tukey's multiple comparisons test (** = *p* < 0.01). (G) rVAR2‐associated fluorescence signal, measured by FCM and expressed in MESF per millilitre, for plasma from one PDAC patient, with corresponding healthy and chABC controls. (H) PDAC and (I) healthy plasma labelled with rVAR2. (J) PDAC sample incubated with chABC prior to rVAR2 labelling. (K) PDAC plasma treated with NP40 after rVAR2 labelling. Scale bars in the main images and insets represent 10 µm and 2 µm, respectively. (L) rVAR2 mean fluorescence intensity of three representative images per plasma sample from five PDAC patients and corresponding healthy controls measured by FM. (M) rVAR2‐associated fluorescence signal, measured by FCM and expressed in MESF per millilitre, for plasma from five PDAC patients and corresponding healthy controls. (N) Log‐transformed patient‐to‐healthy rVAR2‐associated signal ratios for FM and FCM. Line at median.

To investigate the presence of ofCS on tdEVs in pancreatic cancer, we first evaluated the rVAR2 labelling of the pancreatic cancer PANC‐1 cells and their EVs. rVAR2 labelling of PANC‐1 cells exhibited notable variability, with some cells not displaying ofCS (Figure S3D‐F). Figure 5A,D,E shows rVAR2^+^ EVs derived from PANC‐1 cells, which were notably less abundant than CD81^+^ EVs. Treatment with chABC and NP40 reduced the rVAR2 signal, as measured by both FM and FCM (Figure 5B‐E).

### 3.6 ofCS‐Modified Extracellular Vesicles Are Present in the Plasma of Pancreatic Cancer Patients

To validate whether rVAR2 can detect ofCS‐modified tdEVs in cancer patients, we analysed plasma samples from PDAC patients (*n* = 5, Table ST1) using Airyscan FM and FCM. To assess the labelling specificity, pooled plasma collected from four healthy donors was processed alongside patient samples. Patient and healthy samples were handled in pairs, and the total particle concentrations, including both stained and unstained particles, are shown in Table ST3.

The combined imaging and FCM analysis showed a clear difference in labelling intensity and concentration of rVAR2^+^ particles between the plasma from cancer patients and the healthy controls as shown for one patient in Figure 5F‐I. Treatment of PDAC plasma with chABC reduced the number of rVAR2^+^ particles, but not the number of CD81^+^ particles (Figure 5F,G,J). Additionally, treatment of the rVAR2^+^ PDAC plasma with NP40 reduced the number of particles labelled with rVAR2 and anti‐CD81 (Figure 5F,K). Figure 5L‐M illustrates that the plasma of all but one PDAC patient contained detectable concentrations of rVAR2‐binding particles, while these concentrations were markedly reduced in the plasma of healthy controls. Additionally, the log‐transformed rVAR2 signal ratios (rVAR2_Patient_/rVAR2_Healthy_) were greater than 0 for four out of five patients (Figure 5N). These results yielded *p* = 0.031 for FM and *p* = 0.068 for FCM. Furthermore, FM images of the NP40 controls showed a decrease in the number of particles labelled with rVAR2 (Figure S8). In cases where a sufficient patient plasma was available, a chABC control was included, demonstrating a significant reduction in the rVAR2 fluorescence signal (Figure S8I,J,S,T). To ensure that rVAR2 background staining in healthy plasma was not influenced by donor age, we analysed plasma pooled from age‐matched healthy donors (>50 years old). Background rVAR2 signal remained low (Figure S9), consistent with our earlier findings using plasma from a mixed‐age donor pool.

To further assess rVAR2 labelling in PDAC patient plasma, we analysed additional biobank plasma samples from PDAC patients. Since these samples were collected in citrate tubes and the first measurements were done with EDTA plasma, we compared healthy EDTA and citrate plasma to evaluate potential differences in background fluorescence. As shown in Figure S10, citrate plasma exhibited lower background rVAR2 signal, supporting its use for subsequent analyses. Based on this, we examined six additional PDAC patient plasma samples collected in citrate tubes. These were analysed in three independent experimental sets, each including a pooled healthy citrate plasma sample as a control reference. As shown in Figure S11, three of the six PDAC patients exhibited higher rVAR2 signal compared to the healthy control, while the other three did not.

## 4 Discussion

This study shows that ofCS, a malignancy‐associated glycosaminoglycan modification, is present on tdEVs and can be targeted using rVAR2, a recombinant lectin that binds ofCS. The coupling of rVAR2 with a fluorophore enabled the identification of EVs from cancer cells in vitro and tdEVs spiked into healthy plasma. Furthermore, we showed that ofCS is retained on tdEVs following epithelial‐to‐mesenchymal transition. Finally, in a small cohort of patients with stage III and IV PDAC, we detected the presence of ofCS^+^ EVs in some but not all patients. These findings support ofCS as a potential marker for tdEVs and suggest that rVAR2 can be helpful in identifying and characterizing tdEVs.

We used several orthogonal techniques for single EV detection to validate the findings, and minimise the risk of methodological biases. These techniques included super‐resolution FM either of uncaptured or selectively captured EVs, and calibrated FCM. We analysed EVs from cancer cell lines, EV‐spiked plasma from healthy donors and patient plasma. Furthermore, to control for day‐to‐day variations, we included healthy controls alongside patient samples to ensure procedural consistency and reliability of our reagents and processes. The results obtained were consistent across the different methods, supporting the robustness of the data.

To confirm the specificity of the rVAR2 binding to ofCS on tdEVs, we employed multiple controls. ChABC and rVAR2 mutant controls demonstrated a clear decrease in the signal and number of particles labelled with rVAR2, validating the specific binding of rVAR2 to CS. Detergent treatment controls showed a decrease in rVAR2 signal confirming the labelling of EVs. Procedural controls including reagents in the buffer showed minimal background due to reagents.

We showed that ofCS is retained on tdEVs following epithelial‐to‐mesenchymal transition. This indicates that tdEV detection is not limited to EVs from epithelial‐like tumour cells, thereby broadening the clinical applicability of ofCS+ EV detection. Additionally, we identified ofCS on EVs derived from lung, pancreatic, colorectal, ovarian, glioblastoma and melanoma cancer cell lines, albeit at different levels, while EVs from non‐malignant cell lines generally exhibited lower rVAR2 staining. EVs from (non‐metastatic) MCF‐7 cells, which retain several characteristics of differentiated mammary epithelium, did not show detectable rVAR2 staining. Notably, low‐level rVAR2 binding was observed in some non‐malignant EVs, particularly from HMEC cells. This may reflect characteristics of the immortalisation process (by serial passaging and transduction with LXSN‐based retroviruses carrying hTERT and a mutant CDK4(R24C)) or high passage numbers, which could induce low aberrant expression of ofCS or induce off‐target binding by expression of chondroitin sulphate protein modifications different from ofCS.

The presence of ofCS on EVs, as detected specifically with rVAR2 or newly developed anti‐ofCS (C9) antibodies, is a novel diagnostic investigation. However, several proteoglycans, previously shown to display ofCS, have been investigated as EV biomarkers. For instance, a paper from 2019^31^ found syndecan‐1 (SDC‐1) on the surface of EVs in plasma from high‐grade glioblastoma multiforme (GBM) patients, where SDC‐1 was used to distinguish high‐grade from low‐grade GBM (Chandran et al. 2019). Furthermore, syndecan‐4 was also found on EVs in gastric cancer (Poças et al. 2023). SDC‐1 and SDC‐4 are both known as ofCS‐carrying proteoglycans in cancer (Vidal‐Calvo et al. 2024; Bang‐Christensen et al. 2019; Seiler et al. 2017). Additionally, proteoglycans have been reported to be among the most common EV‐proteins on tdEVs from multiple cancers (Hoshino et al. 2020).

Two reports have demonstrated the utility of cell‐free ofCS as a cancer biomarker in plasma and urine (Zhang et al. 2023; Clausen et al. 2020). Zhang et al. quantified ofCS‐modified proteoglycans in plasma, showing elevated levels across multiple cancer types and suggesting their potential for pan‐cancer early detection. Clausen et al. developed a dot‐blot assay to detect ofCS in urine, particularly in bladder cancer, and demonstrated its association with tumour burden. In contrast to these reports (Zhang et al. 2023; Clausen et al. 2020), which measured total cell‐free ofCS regardless of origin, our study specifically detects and analyses EVs to investigate the presence of ofCS on tdEVs. This approach allows us to exclude soluble ofCS‐proteoglycans and directly demonstrate that a portion of the circulating ofCS signal may originate from tumour EVs. While it remains to be determined which approach is more diagnostically informative, our findings provide mechanistic insight into the source of cell‐free ofCS and support the potential of EV‐associated ofCS as a biomarker.

One limitation of this study is the persistence of rVAR2+ particles after NP‐40 treatment. While the rVAR2 binding is confirmed to be CS‐specific by the decline in staining after treatment with chABC and reagent controls, showing a virtual absence of the rVAR2 signal, the detergent lysis did not fully abolish the signal. Thus, the residual signal after NP‐40 treatment may originate from incomplete lysis of rVAR2^+^ EVs. The EV fragments may still retain labelled ofCS epitopes, and their fluorescence intensity may exceed the detection threshold of our detection systems. Unlike tetraspanins, which are present in lower densities, ofCS is displayed across multiple proteins, resulting in higher epitope abundance and potentially detectable residual fluorescence after partial lysis. Finally, we cannot rule out the possibility that in the presence of rVAR2, multimerisation of free circulating ofCS‐modified proteoglycans occurs (Zhang et al. 2023).

In our proof‐of‐concept study, we detected rVAR2^+^ EVs in PDAC patients, with four out of five patients exhibiting higher rVAR2 staining than healthy controls. A limitation of our study is that PDAC biobank samples were not filtered through a 0.8 µm filter, potentially co‐isolating platelets and erythrocyte ghosts. However, this is unlikely to account for the observed differences, as rVAR2+ particles detected by fluorescence microscopy and flow cytometry were significantly smaller than platelets and erythrocyte ghosts, which are in the micrometre range. To further validate our findings and expand the patient pool, we analysed additional PDAC samples from a second cohort, in which three out of six patients showed higher rVAR2 staining compared to healthy controls. While these results support the potential of ofCS as a marker for tdEVs in PDAC, the variability observed across cohorts highlights the need for larger sample sizes and further clinical validation. Future studies should also include a broader range of tumour stages, cancer types, and patients with benign lesions to evaluate the general applicability of ofCS as a pan‐cancer tdEV marker.

Our findings indicate that while rVAR2 labelling successfully identifies tdEVs, a detectable signal is also present in healthy plasma, which, in some cases, differed between measurement days. This difference may be attributed to the manual processing of the samples, for example, during rVAR2‐fluorophore conjugation and aggregate removal. Additionally, the rVAR2 signal in healthy plasma may result from off‐target or low‐affinity binding of rVAR2, a limitation of our study. To address this, we tested a newly developed antibody fragment (C9) against ofCS (Vidal‐Calvo et al. 2024). We observed that C9 staining of healthy plasma has a lower background signal than rVAR2, while still being capable of binding to ES‐2‐derived EVs that were spiked into plasma, as shown in Figure S12. These results further validate the detection of tdEVs containing ofCS and suggest that C9 may be beneficial for improving specificity in future studies.

Our study shows the presence of ofCS on tdEVs derived from cancer cell lines and also in the plasma of PDAC patients. We present a novel method for detecting tdEVs by targeting ofCS with rVAR2 or C9 antibody. This approach can potentially be used to detect, isolate and characterize tdEVs not only from PDAC, but also from other tumour types, which can be used to explore tdEV function and biomarker potential in cancer. The utility of tdEVs as diagnostic biomarkers and indicators of therapeutic response has been previously highlighted (Hoshino et al. 2020, Casanova‐Salas et al. 2024). Our findings suggest that ofCS‐modified tdEVs are part of the pool of circulating EVs in cancer patients. Consequently, detecting and isolating of tdEVs by targeting ofCS could be explored for early‐stage cancer detection and treatment management. Of note, while our manuscript was in revision and in line with our findings, Zhao et al. (2025) reported diagnostic potential of ofCS^+^ EVs in PDAC plasma using rVAR2‐conjugated bead‐based flow cytometry preceded by ultracentrifugation.

## Author Contributions

**Agustin Enciso‐Martinez**: Conceptualization (lead), formal analysis (lead), investigation (lead), methodology (lead), project administration (lead), resources (lead), supervision (lead), visualization (equal), writing ‐ original draft (lead), writing – review and editing (lead). **Caroline Løppke**: Conceptualization (lead), investigation (lead), methodology (lead), project administration (equal), writing – original draft (equal), writing – review and editing (lead). **Joyce J. Koene**: Investigation (equal), Methodology (equal), writing – review and editing (equal). **Jade Ebbelaar**: Investigation (equal), methodology (equal), writing – review and editing (equal). **Meike van der Geest**: Investigation (equal), methodology (equal), writing – review and editing (equal). **Mandy Los**: Investigation (equal), methodology (equal), writing – review and editing (equal). **Emma P. E. M. Boerrigter**: Investigation (equal), Methodology (equal), writing ‐ review and editing (equal). **Robert Dagil**: Resources (equal), writing ‐ review and editing (equal). **Tobias Gustavsson**: Resources (equal), writing ‐ review and editing (equal). **Elena E. Vidal‐Calvo**: Resources (equal), writing ‐ review and editing (equal). **Ton G. van Leeuwen**: writing ‐ review and editing (equal). **Roman I. Koning**: Investigation (equal), Methodology (equal), writing ‐ review and editing (equal). **Nick van Es**: Resources (equal), writing ‐ review and editing (equal). **Ali Salanti**: Funding acquisition (equal), resources (equal), writing ‐ review and editing (equal). **Edwin van der Pol**: Funding acquisition (lead), writing ‐ original draft (lead), writing ‐ review and editing (equal). **Rienk Nieuwland**: Funding acquisition (equal), writing ‐ original draft (lead), writing ‐ review and editing (lead). **Peter ten Dijke**: Conceptualization (lead), funding acquisition (lead), project administration (lead), resources (lead), supervision (lead), writing ‐ original draft (lead), writing ‐ review and editing (lead).

## Conflicts of Interest

M.Ø.A. and A.S. are co‐founders and shareholders in VAR2 Pharmaceuticals and VarCT Diagnostics, holding the patent for developing and commercialising the rVAR2 technology for diagnostic purposes. The antibodies are subject to a patenting process owned by VAR2 Pharmaceuticals. E.v.d.P. is co‐founder and shareholder of Exometry B.V., Amsterdam, the Netherlands.

## Supporting information




**Supplementary Materials**: jev270106‐sup‐0001‐SuppMat.docx

## Data Availability

The data generated in this study are available upon request from the corresponding author. Other data generated in this study are available within the article and its supplementary data files. Flow cytometry data details are reported in MIFlowCyt‐EV.
