# Supplementary material for: Targeting Oncofoetal Chondroitin Sulphate Allows Identification of Tumour‐Derived Extracellular Vesicles
Source: J Extracell Vesicles. 2025 Jun 17;14(6):e70106. doi: 10.1002/jev2.70106 (PMC12173530; doi:10.1002/jev2.70106)
Supplement: Supplementary file 1 — Supplementary Materials: jev270106‐sup‐0001‐SuppMat.docx [file JEV2-14-e70106-s001.docx]

**Oncofetal chondroitin sulfate allows identification of tumor-derived extracellular vesicles**

Agustin Enciso-Martinez*(1,2,3), Caroline Løppke (4), Joyce J. Koene^†^ (1), Jade Ebbelaar^†^ (3), Meike van der Geest (1), Mandy Los (3), Emma Boerrigter (1), Robert Dagil (4), Tobias Gustavsson (4,5), Elena E. Vidal-Calvo (4), Ton G. van Leeuwen (2), Roman I. Koning (6), Nick van Es (7,8), Ali Salanti (4), Edwin van der Pol^‡^ (2,3), Rienk Nieuwland^‡^ (3), Mette Ø. Agerbæk^‡^* (4,9) and Peter ten Dijke^‡^ (1)

1. Oncode Institute, Department of Cell and Chemical Biology, Leiden University Medical Center, Leiden, The Netherlands
2. Amsterdam UMC, University of Amsterdam, Biomedical Engineering & Physics, Meibergdreef 9; Cancer Center Amsterdam, Imaging and Biomarkers, Amsterdam, The Netherlands
3. Amsterdam UMC, University of Amsterdam, Laboratory of Experimental Clinical Chemistry; Laboratory Specialized Diagnostics & Research, Department of Laboratory Medicine, Meibergdreef 9, Amsterdam, The Netherlands
4. Centre for translational Medicine and Parasitology at Department of Immunology and Microbiology, Faculty of Health and Medical Sciences, University of Copenhagen, Copenhagen, Denmark
5. VAR2 Pharmaceuticals ApS, Frederiksberg, Denmark
6. Electron Microscopy Facility, Department of Cell and Chemical Biology, Leiden University Medical Center, Leiden, The Netherlands
7. Department of Vascular Medicine, Amsterdam UMC location University of Amsterdam, Amsterdam, Netherlands
8. Amsterdam Cardiovascular Sciences, Pulmonary Hypertension & Thrombosis, Amsterdam, The Netherlands
9. VarCT Diagnostics, Frederiksberg, Denmark

**Correspondence to:** Agustin Enciso-Martinez, email: [a.enciso_martinez@lumc.nl](mailto:a.enciso_martinez@lumc.nl)

Mette Ø. Agerbæk, email: [mettea@sund.ku.dk](mailto:mettea@sund.ku.dk)

^†^These authors contributed equally

**Supplementary Figures**

**
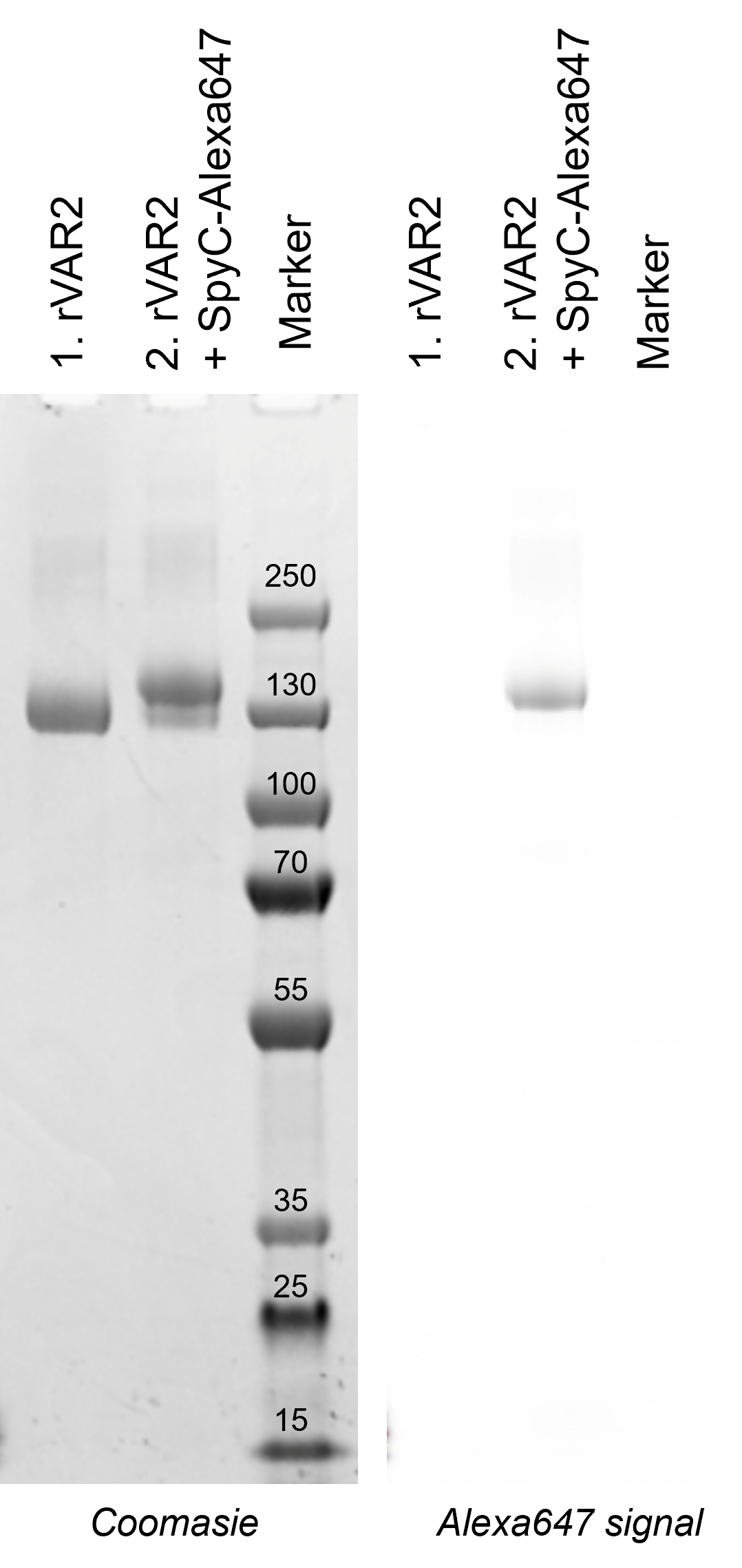
**

**Figure S1.** Alexa647-Spycatcher binding to rVAR2 was evaluated by sodium dodecyl sulfate polyacrylamide gelelectrophoresis (PAGE) (SDS-PAGE) .

**
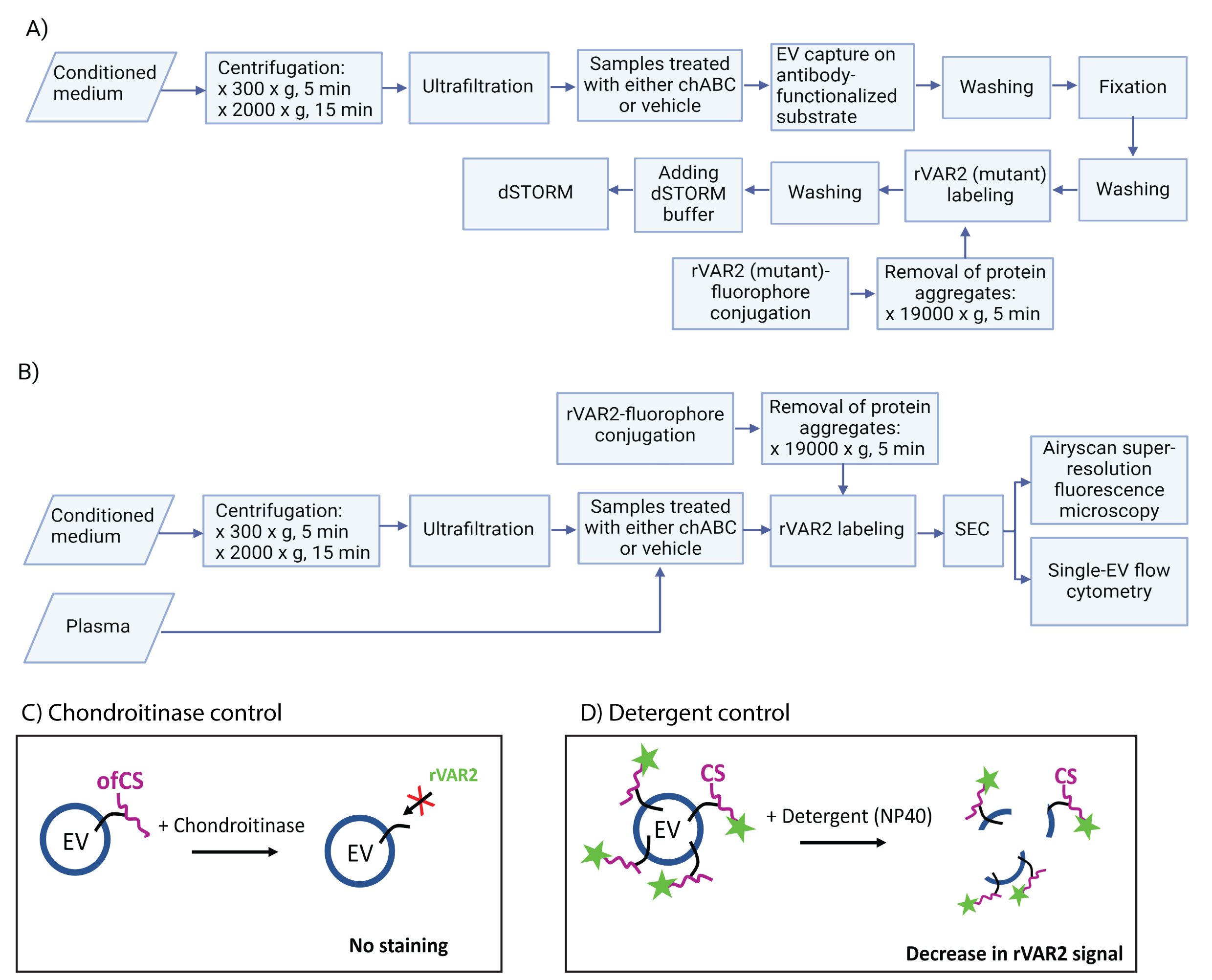
**

**Figure S2. Overview of sample processing and analysis.** (**A**) rVAR2 labeling of A549-derived EVs captured on a substrate funtionalized with CD9, CD63 and CD81 antibodies, and imaged using dSTORM. (**B**) rVAR2 labeling of EVs from cancer cell lines and patient plasma, analyzed using Airyscan fluorescence microscopy and flow cytometry. SEC: size exclusion chromatography. (**C**) Schematic of chondroitinase (chABC) control for rVAR2 labeling specificity. (**D**) Schematic of detergent control for EV labeling specificity.

**
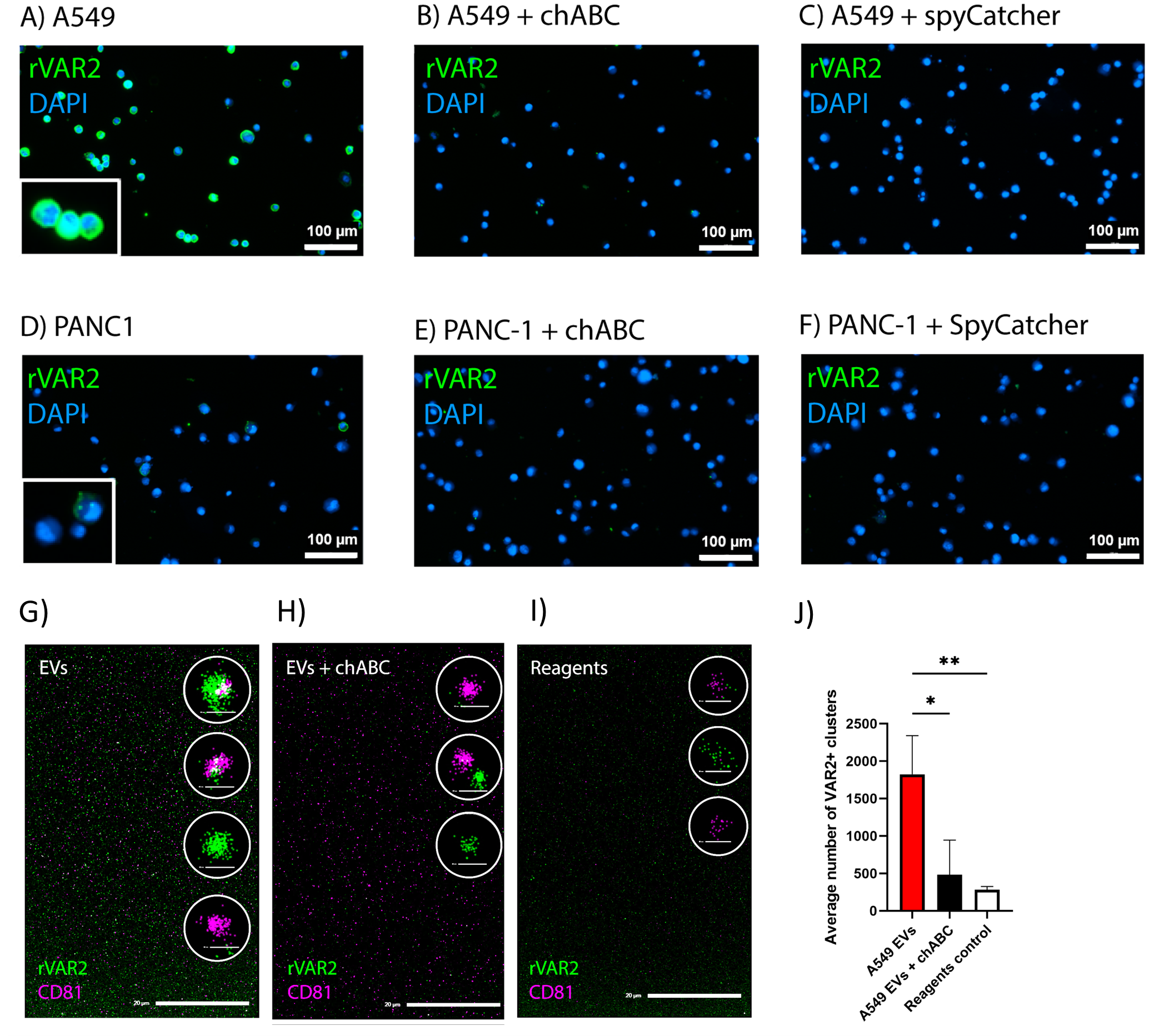
**

**Figure S3. rVAR2 staining of A549 and PANC1 cells using a recombinant version of the malaria parasite protein VAR2CSA (rVAR2).** (**A**) A549 and (**D**) PANC-1 cancer cells stained with rVAR2. (**B**) A549 and (**E**) PANC-1 cells were treated with chondroitinase ABC enzyme (chABC) prior to rVAR2 staining. (**C**) A549 and (**F**) PANC-1 cells stained with SpyCatcher-AF647 only (no rVAR2). The nucleus is stained with 4′,6-diamidino-2-phenylindole (DAPI; blue). Images were acquired using fluorescence microscopy. (**G-J**) Phosphatidyl serine (PS)-based captured of A549-derived EVs stained with rVAR2 and imaged using dSTORM. (**G**) A549-derived EVs stained with rVAR2. (**H**) A549-derived EVs treated with chABC prior to rVAR2 staining. (**I**) Procedural control (no EVs, only reagents). The insets show typical clusters of detected single molecules. The scale bar represents 20 µm in the image overviews, and 80 nm in the insets. (**J**) Average rVAR2+ clusters per image (n=3 images per condition). One-way ANOVA with Tukey’s multiple comparisons test (* = *p* < 0.05, ** = *p* < 0.01).


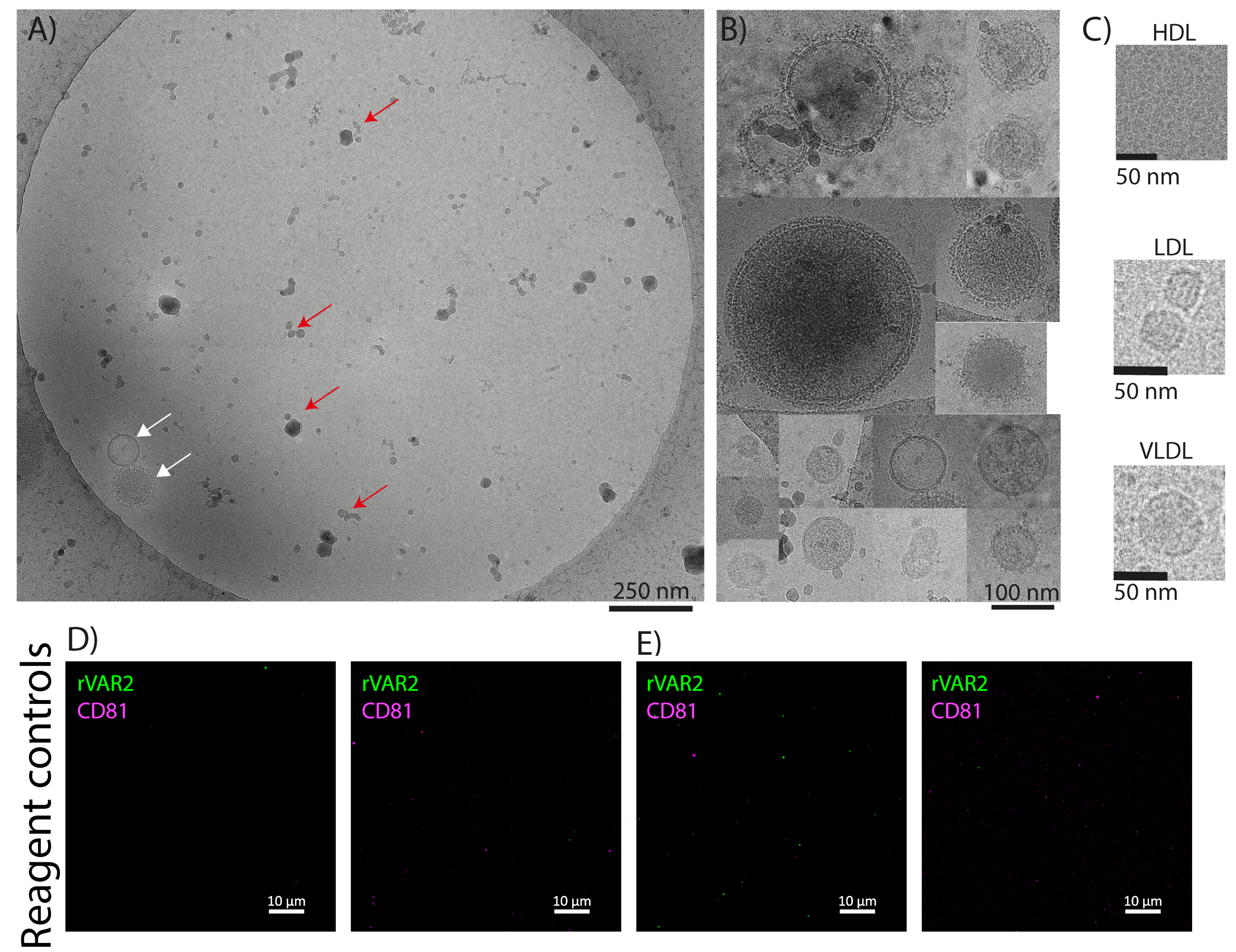


**Figure S4. Cryo-electron microscopy (cryo-EM) images of A549-derived extracellular vesicles (EVs) and reagent controls.** High-throughput cryo-EM was performed on the EV samples to evaluate their presence and sample quality. Automatic image acquisition was performed to prevent operator-dependent bias, reflecting, as such, the overall sample composition. One hundred thirty-nine cryo-EM images were analyzed and compared with images of plasma-purified lipoproteins (**C**) to exclude their presence in the EV preparations. (**A**) A representative image of a hole in the EM grid indicating EVs with arrows, and ice contamination with red arrows. The scale bar represents 250 nm. (**B**) Representative images of EVs. The scale bar represent 100 nm. (**C**) Representative cryo-EM images of commercially available HDL (top), LDL (middle) and VLDL (bottom) imaged for comparison. (**D-E**) Reagent controls (dPBS + 3% filtered FBS) stained with rVAR2-AF647 and CD81-AF488 showing a virtual absence of fluorescence signals. Representative images of two independent experiments.

**
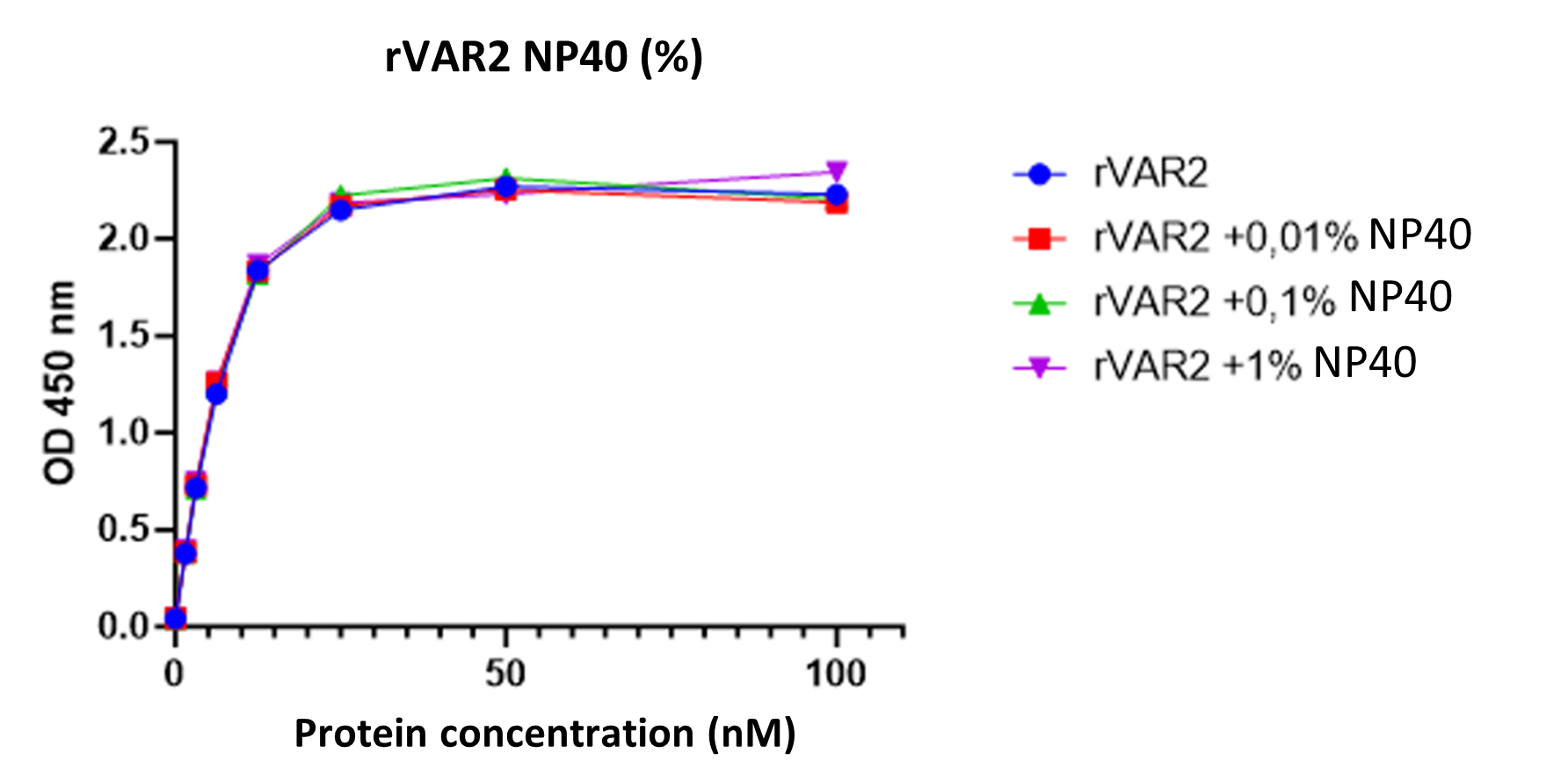
**

**Figure S5. Nonidet P-40 (NP40) detergent does not interfere with the binding capabilities of rVAR2 to chondroitin sulfate (CS).** ELISA assay: Optical density (OD) at 450 nm was measured for different rVAR2 concentrations in the presence of different concentrations of NP40.


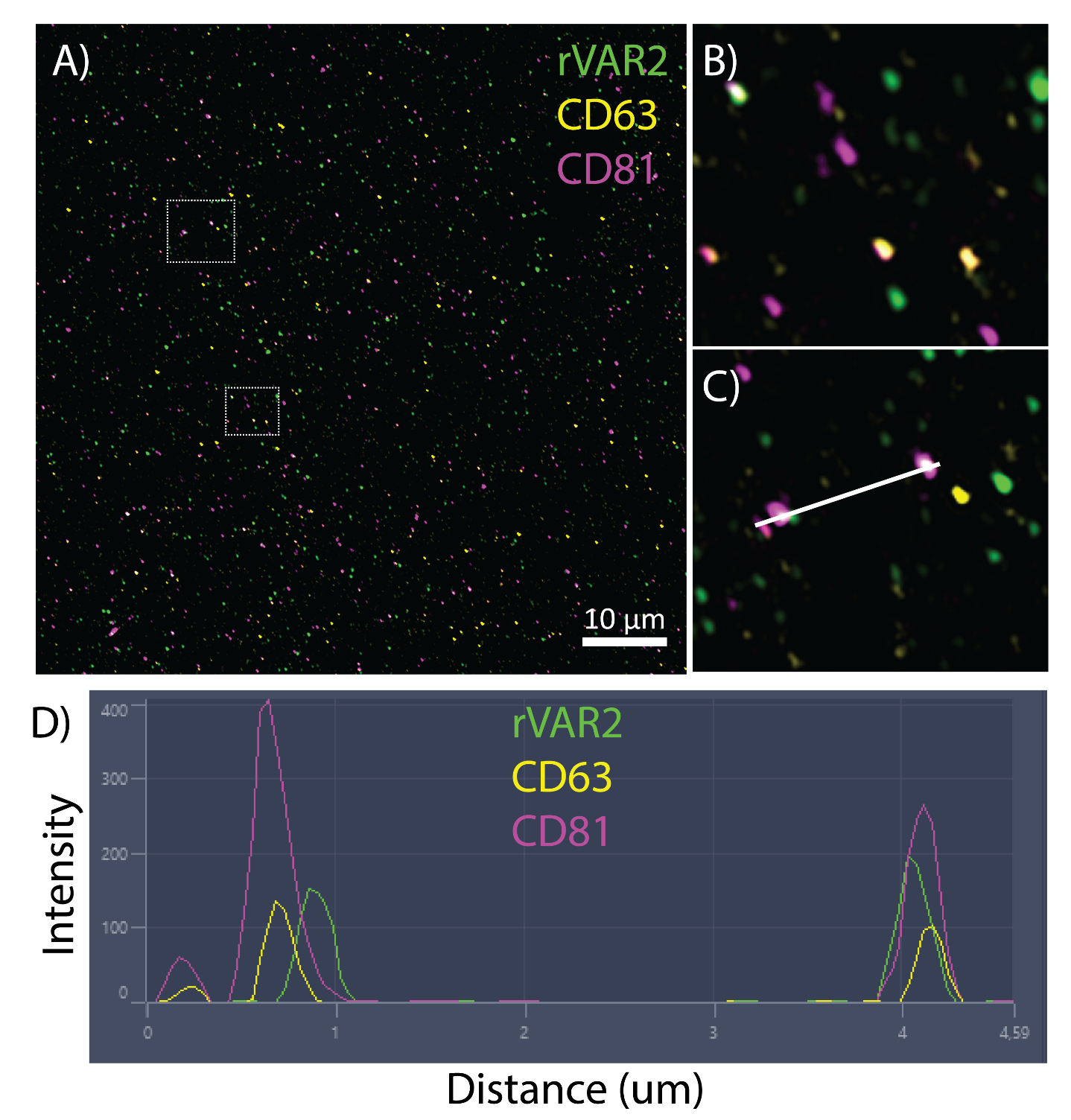


**Figure S6.** **Colocalization of CD63, CD81, and rVAR2 on A549-derived EVs.** (**A**) Representative fluorescence microscopy image of EVs stained for CD63, CD81, and rVAR2. (**B-C**) Zoom-in views from panel A highlighting individual EVs. (**D**) The line profile of fluorescence intensity along the indicated region in C shows colocalization of markers in some EVs.


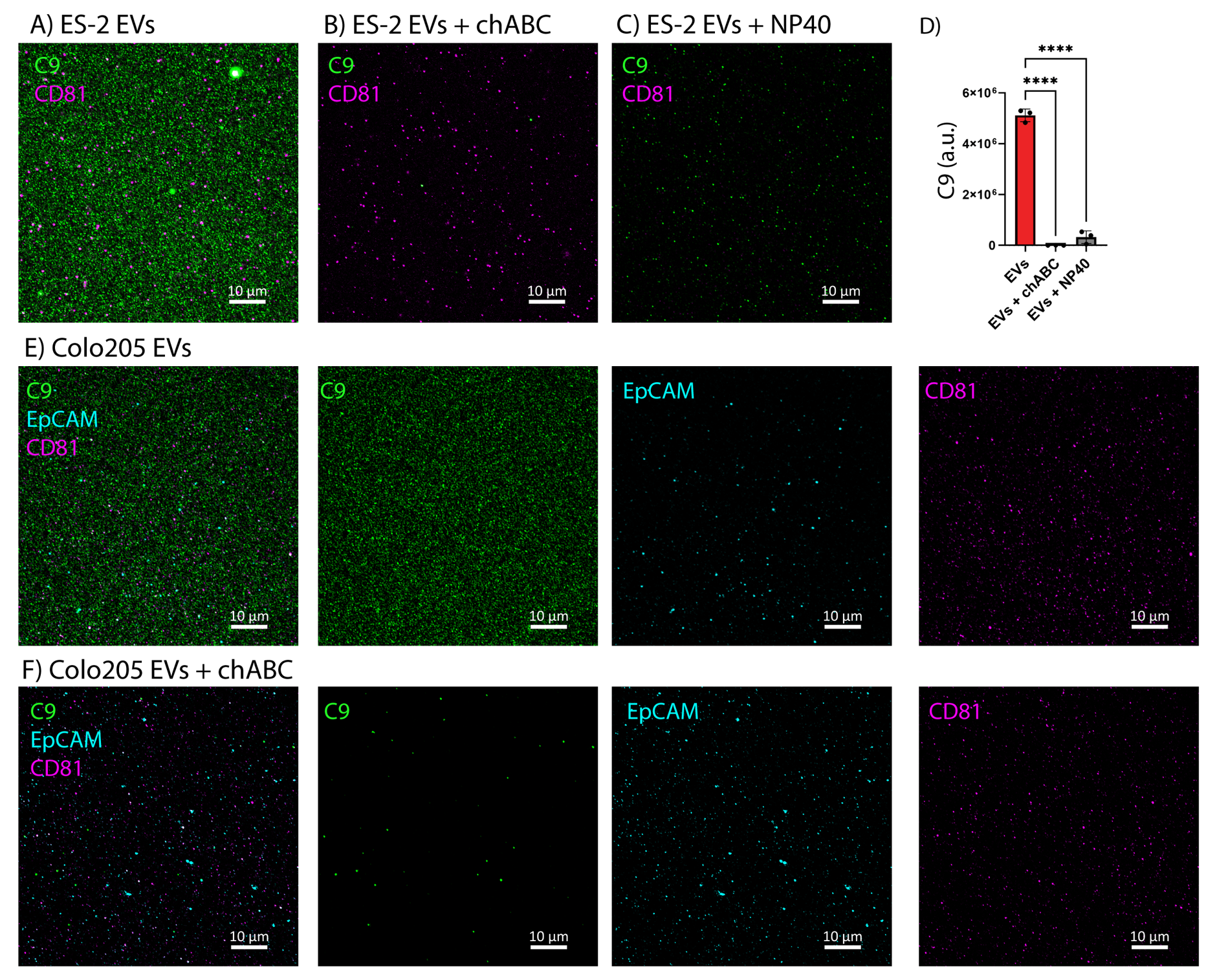


**Figure S7. Anti-ofCS single-chain variable fragment C9 labeling of ES-2- and Colo205-derived EVs as evaluated by Airyscan fluorescence microscopy.** (**A**) ES-2-derived EVs labeled with C9 and CD81, (**B**) ES-2-derived EVs treated with chABC before labeling, and (**C**) ES-2-derived EVs treated with NP40 after labeling. (**D**) Mean C9 fluorescence intensity of 3 representative images per condition for ES-2-derived EVs. Statistical analysis was performed using one-way ANOVA with Tukey’s multiple comparisons test (**** = *p* < 0.0001). Error bars represent standard deviation. (**E**) Colo205-derived EVs labeled with C9, EpCAM and CD81. (**F**) Colo205-derived EVs treated with chABC before labeling.

**
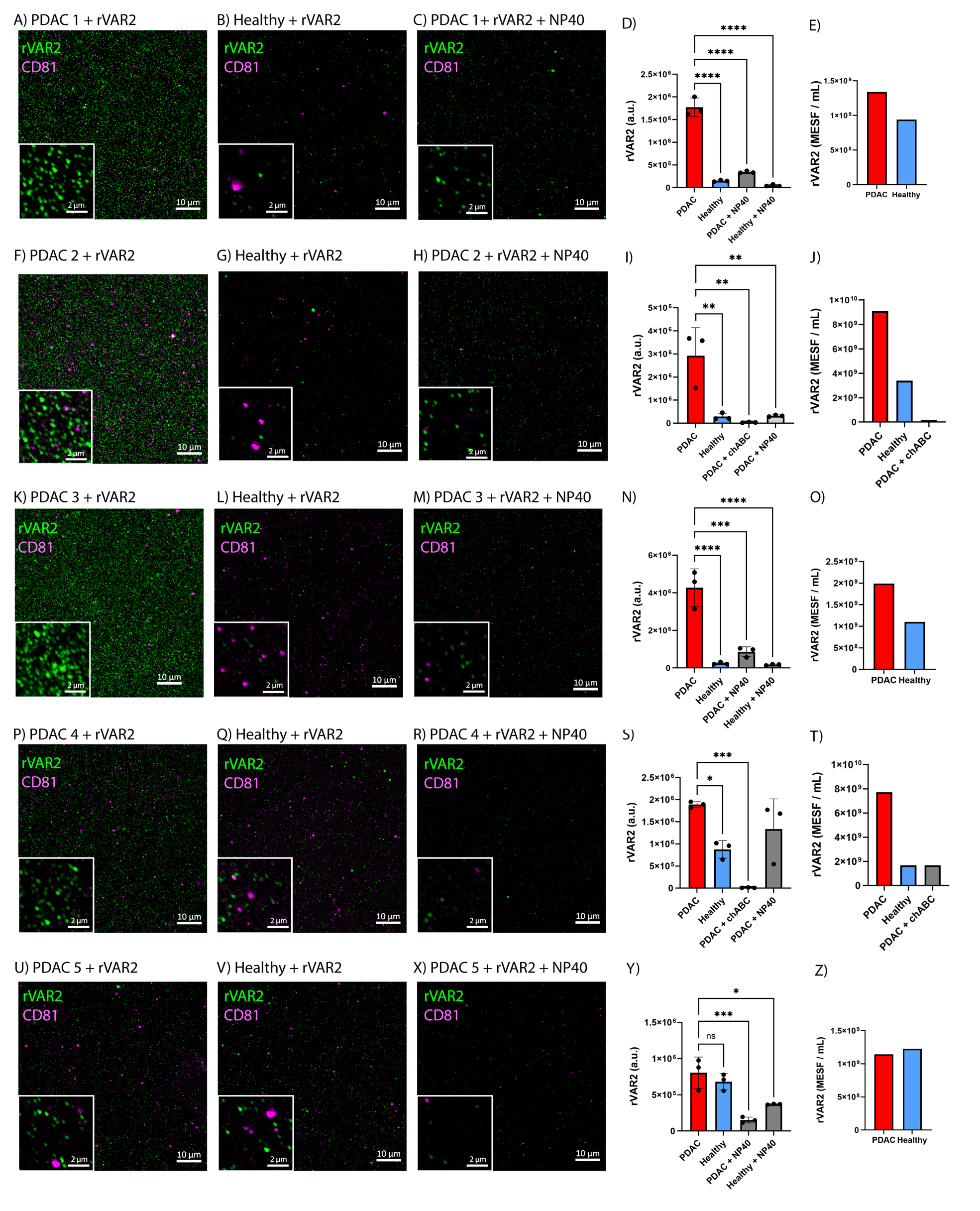
**

**Figure S8.** **rVAR2 staining of plasma from a cohort of five PDAC patients using rVAR2, and evaluated using Airyscan fluorescence microscopy and flow cytometry.** Each row represents a plasma sample from an individual PDAC patient, processed in parallel with plasma from a pooled sample of four healthy donors. For patient 1 pooled SEC fractions 2-4 are shown. Columns (1-3) show representative Airyscan fluorescence microscopy images of rVAR2-labeled plasma samples from PDAC patients, healthy individuals, and PDAC samples treated with 1% NP40, respectively. Column 4 shows the mean rVAR2 fluorescence intensity of 3 representative images per condition. Statistical analysis was performed using one-way ANOVA with Tukey’s multiple comparisons test (* = *p* < 0.05, ** = *p* < 0.01, *** = *p* < 0.001, **** = *p* < 0.0001). Error bars represent standard deviation. (**I**) and (**S**) bar plots include data from chABC-treated PDAC plasma. Column 5 shows the rVAR2-associated fluorescence signal, measured by FCM and expressed in MESF per mL, for PDAC and healthy plasma, with corresponding chABC controls for (**J**) and (**T**).

**
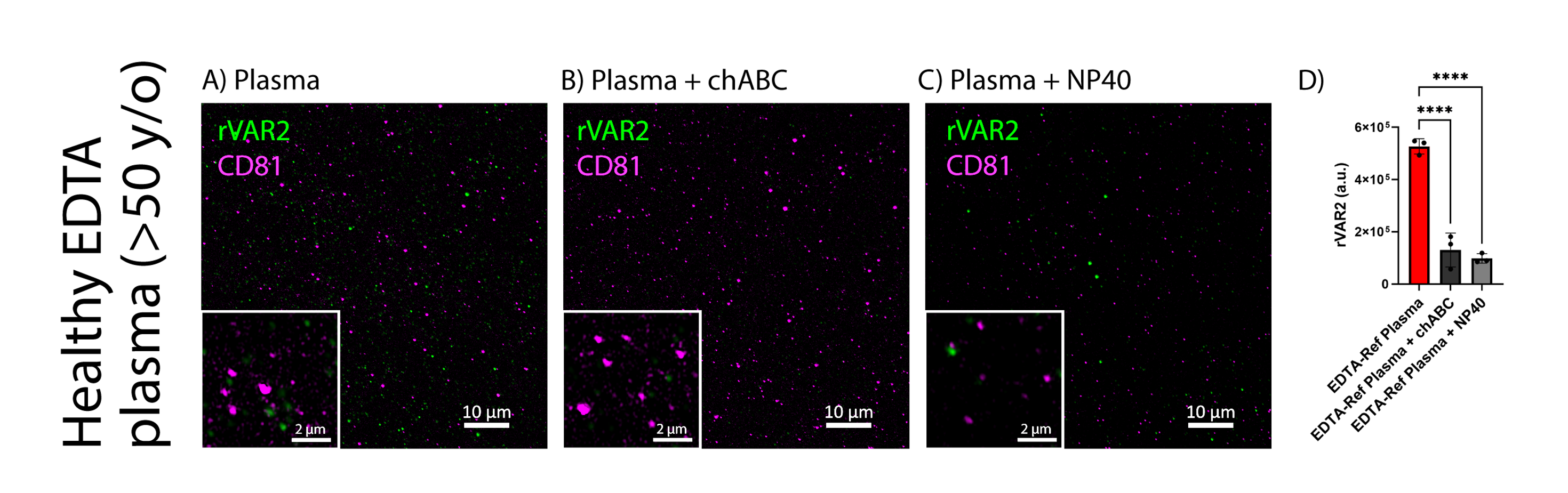
Figure S9. rVAR2 labeling of age-matched healthy plasma (n=4 donors >50 years old) as evaluated by Airyscan fluorescence microscopy.** (**A**) Plasma labeled with rVAR2, (**B**) Plasma treated with chABC before labeling, and (**C**) Plasma treated with NP40 after labeling. (**D**) Mean rVAR2 fluorescence intensity of 3 representative images per condition. Statistical analysis was performed using one-way ANOVA with Tukey’s multiple comparisons test (**** = *p* < 0.0001). Error bars represent standard deviation.

**
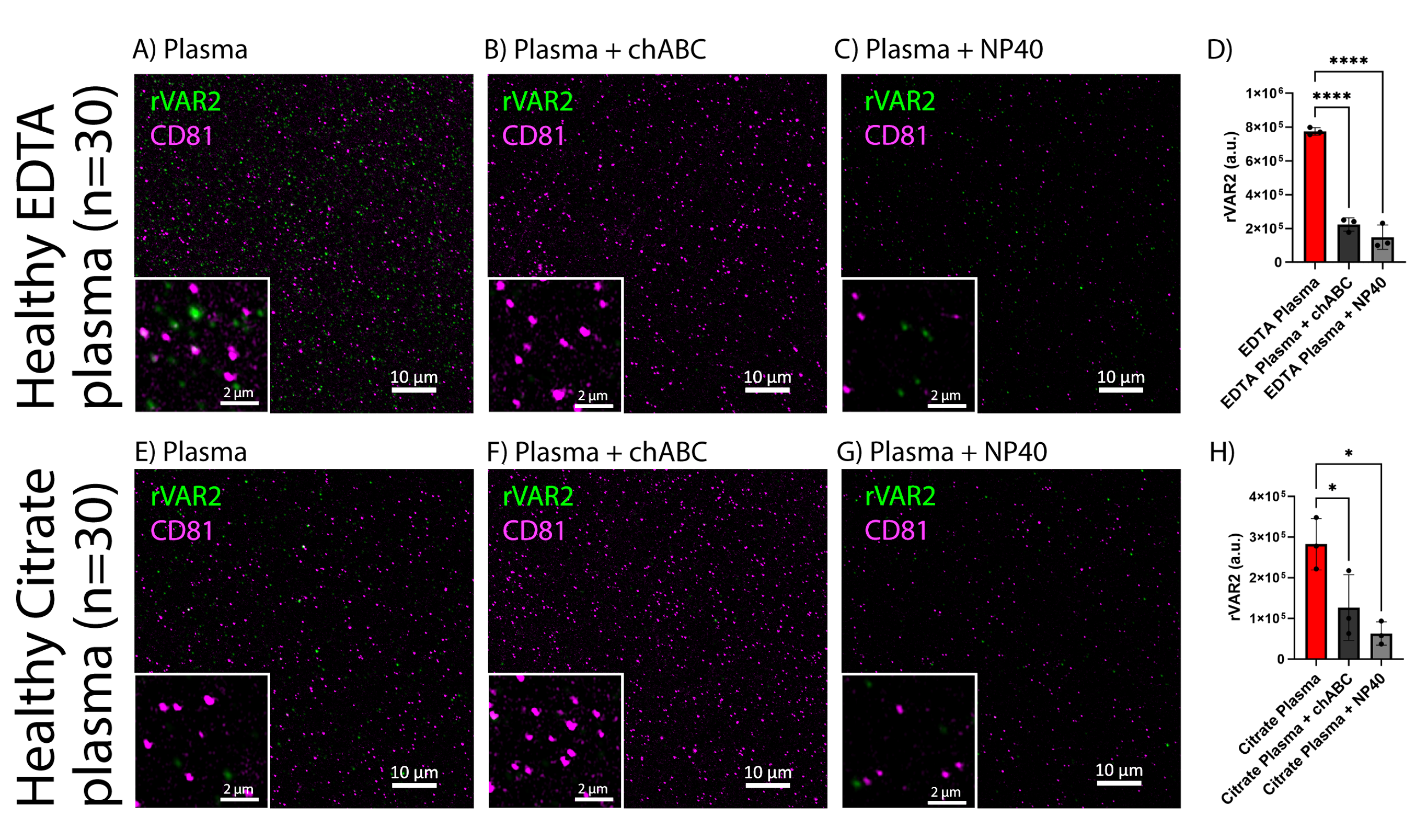
**

**Figure S10. rVAR2 labeling of healthy plasma (n=30 donors) as evaluated by Airyscan fluorescence microscopy.** (**A**) EDTA plasma labeled with rVAR2, (**B**) EDTA plasma treated with chABC before labeling. (**C**) EDTA plasma treated with NP40 after labeling. (**D**) Mean rVAR2 fluorescence intensity of 3 representative images per condition for EDTA plasma. (**E**) Citrate plasma labeled with rVAR2, (**F**) Citrate plasma treated with chABC before labeling. (**G**) Citrate plasma treated with NP40 after labeling. (**D**) Mean rVAR2 fluorescence intensity of 3 representative images per condition for citrate plasma. Statistical analysis was performed using one-way ANOVA with Tukey’s multiple comparisons test * = *p* < 0.05, ** = *p* < 0.01, *** = *p* < 0.001, **** = *p* < 0.0001). Error bars represent standard deviation.

**
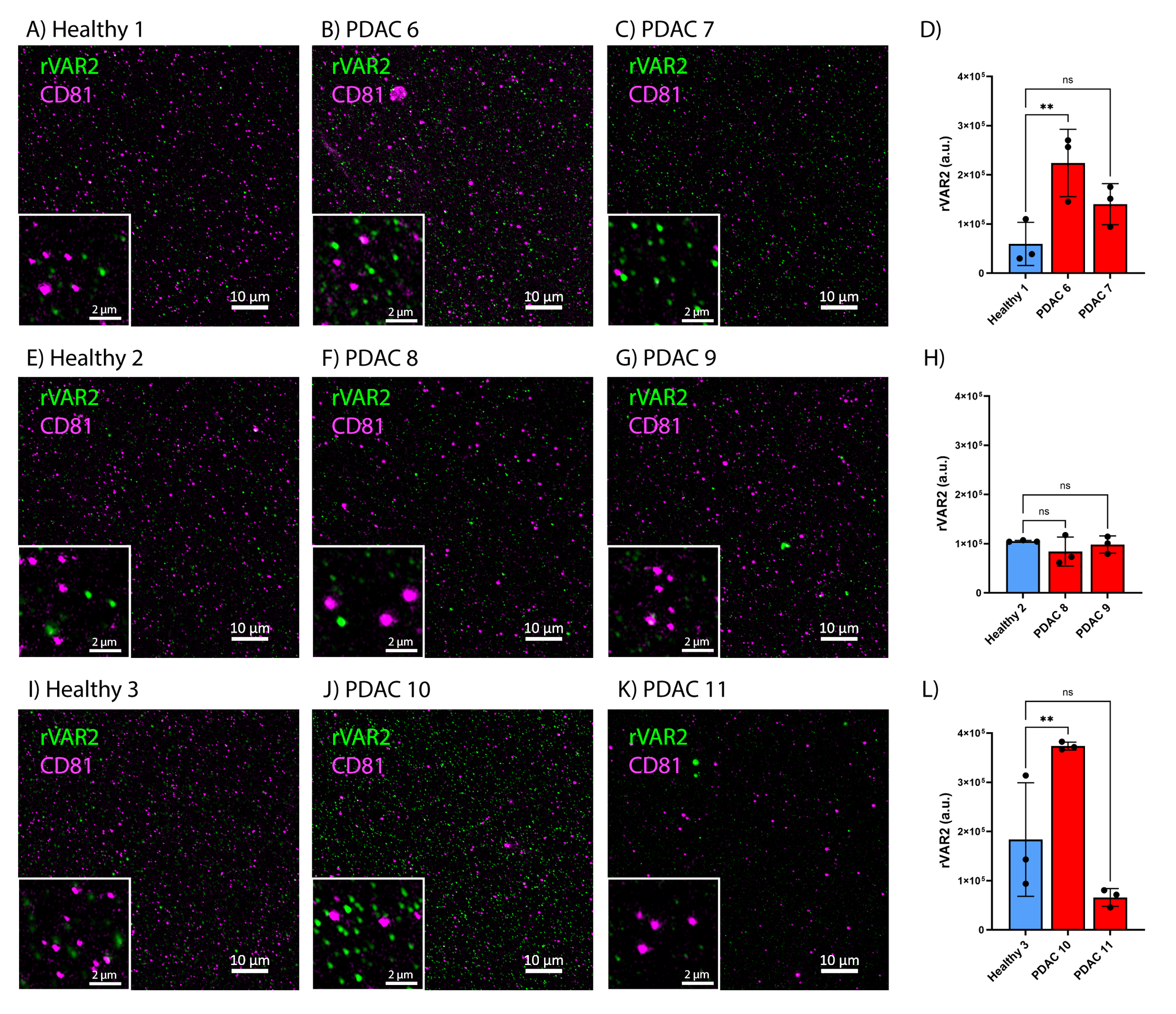
**

**Figure S11. rVAR2 labeling of PDAC and healthy plasma as evaluated by Airyscan fluorescence microscopy.** (**A,E,I**) Healthy citrate pool plasma (n=30 donors) labeled with rVAR2, serving as a control across 3 experimental sets. (**B-C, F-G, J-K**) PDAC patient citrate plasma labeled with rVAR2 from 3 independent sets, each including 2 patients. (**D,H,L**) Mean rVAR2 fluorescence intensity of 3 representative images per sample. A threshold of 180 a.u. was applied to account for background. Statistical analysis was performed using one-way ANOVA with Šidák multiple comparisons test (** = *p* < 0.01). Error bars represent standard deviation.

**
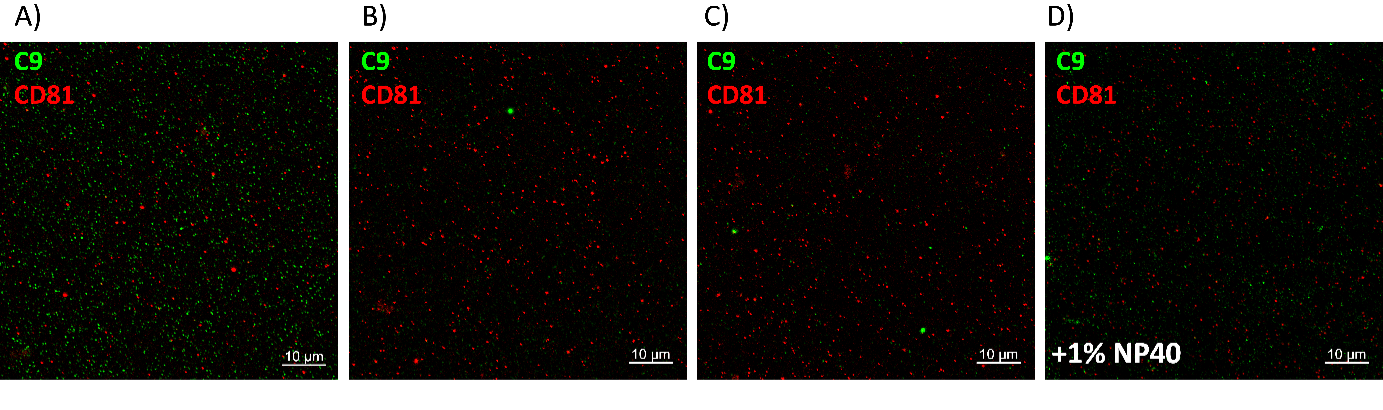
**

**Figure S12. C9 labeling of ES-2-derived EVs spiked into plasma with corresponding negative controls as evaluated by Airyscan fluorescence microscopy.** (**A**) C9 labeling of ES-2-derived EVs spiked into healthy plasma in a 1 to 5 volume ratio. (**B**) C9 labeling of chABC-treated ES-2-derived EVs spiked into healthy plasma. (**C**) C9 labeling of healthy plasma (pool of four donors). (**D**) C9 labeling of ES-2-derived EVs spiked into healthy plasma followed by treatment with NP40. Scale bars represent 10 µm.

**Supplementary Tables**

**Table ST1**. Pancreatic Ductal Adenocarcinoma (PDAC) patient characteristics

| Patient code | Age | Sex | Disease stage | | Metastasis | Figures |
| --- | --- | --- | --- | --- | --- | --- |
| 1 | 67 | Male | IV | Distant metastasis | Liver | Figure 5, S8 |
| 2 | 74 | Male | IV | Distant metastasis | Liver | Figure 5, S8 |
| 3 | 67 | Male | III | Locally advanced |  | Figure 5, S8 |
| 4 | 69 | Male | IV | Distant metastasis | Lung | Figure 5, S8 |
| 5 | 54 | Male | III | Locally advanced |  | Figure 5, S8 |
| 6 | 48 | Male | IV | Distant metastasis | Liver | Figure S11 |
| 7 | 68 | Female | IV | Distant metastasis |  | Figure S11 |
| 8 | 52 | Female | IV | Distant metastasis |  | Figure S11 |
| 9 | 53 | Male | IV | Distant metastasis | Liver | Figure S11 |
| 10 | 65 | Male | III | Locally advanced |  | Figure S11 |
| 11 | 70 | Male | III | Locally advanced |  | Figure S11 |

**Table ST2.** Healthy donor characteristics

| Characteristic | | Cohort 1  (n=4) | Cohort 2  (n=4) | Cohort 3  (n=30) |
| --- | --- | --- | --- | --- |
| Anticoagulant | | EDTA | EDTA | EDTA and Citrate |
| Age | **Mean** | 45.5 | 56.5 | 48.6 |
|  | **Median** | 48 | 56.5 | 52 |
|  | **Range** | 30-56 | 52-61 | 20–64 |
| Gender (M/F) | | 2 Female / 2 Male | 2 Female / 2 Male | 15 Female / 15 Male |
| Figures | | Figure 4,5, S8 | Figure S9 | Figure S10 and S11 (citrate) |

**Table ST3.** Total particle concentration in PDAC and healthy plasma samples as evaluated by flow cytometry

|  | **Total particle concentration (mL^-1^)** | |
| --- | --- | --- |
| **Patient code** | **PDAC patient** | **Healthy control** |
| 1 | 1.3×10^9^ | 1.6×10^9^ |
| 2 | 8.0×10^9^ | 8.0×10^9^ |
| 3 | 3.3×10^9^ | 4.1×10^9^ |
| 4 | 8.8×10^9^ | 2.2×10^9^ |
| 5 | 0.83×10^9^ | 2.8×10^9^ |

**Supplementary Methods**

**rVAR2 staining of cancer cells and their EVs**

Cancer cell staining. Cancer cells at ~70% confluence were detached using Accutase (Gibco^®^, A111-05-01) and washed using Dulbecco’s PBS (DPBS, Sigma, D8537) containing 2% FBS (Thermo Fischer, 10270-106). A portion of the cancer cells were pre-treated with chondroitinase ABC (chABC, 20 µg/mL) in DPBS + 2% FBS for 30 min. (37°C). As a control, untreated cells were incubated for 30 min (37°C) in DPBS + 2% FBS. The cells were washed twice in DPBS + 2% FBS and incubated for 30 min at 4°C with 200 nM rVAR2-AF647 or the SpyCatcher-AF649 diluted in DPBS + 2% FBS with an adjusted NaCl concentration of 300 mM. After staining, the cells were washed twice in DPBS + 2% FBS and fixed in 4% Paraformaldehyde (Alfa Aesar, J61899) for 5 min at room temperature (RT). The cells were then washed in DPBS, and the cell nucleus was stained with 4’,6-diamidino-2-phenylindole (DAPI, 1:1000, Fischer Scientific, D1306) diluted in DPBS. Next, the cells were plated on a microscopy slide, mounted and scanned using the Cytation^TM^ 5 Cell Imaging Multi-Mode Reader (BioTek, 20X magnification, 0.8 N.A). The samples were analyzed using the Gen5 software (BioTek, v3.10).

Phosphatidyl serine (PS)-rich tdEVs capture on a chip. To further validate the rVAR2 binding and phenotypic distribution, tdEVs were captured on a microfluidic chip using PS-affinity capture and then imaged using single-molecule localization microscopy. For this, the EV profiler kit (#EV-MAN-1.0, ONI, Oxford, UK)^1,2^ was used following the instructions of the manufacturer. In brief, a multilane chip was functionalized for phosphatidylserine-based capture followed by a washing step (100 µL of W1). Samples were then loaded to the chip (10 µL per lane) as follows: 1) A549 EVs, 2) A549 EVs pre-treated with chABC and 3) PBS with 3% FBS. After an incubation time of 50 min, all lanes were then washed (100 µL of W1), fixed (20 µL of F1) for 10 minutes and then washed again (100 µL of W1). A blocking buffer (10 µL of N1) was added for 10 minutes, followed by a washing step (100 µL of W1). Next, 10 µL of a mix of 800 nM rVAR2-AF647, 150 mM NaCl and 2 µg/mL CD81-AF488 were added to all lanes. After an incubation time of 50 minutes in the dark, all lanes were washed (100 µL of W1), fixed for 5 min (20 µL of F1) and washed again (100 µL of W1). Right before imaging with direct stochastic optical reconstruction microscopy (dSTORM), the buffer in the channels was exchanged for the dSTORM buffer.

**Cryo-Electron Microscopy (cryo-EM) of EVs**

A549-derived EVs were isolated using workflow S1-B with the exception of labeling. Quantifoil 2/2 electron microscopy grids were glow discharged in 0.2 mbar air, at 25 mA and for 30 seconds using an Easyglow (Pelco). A 3 µl droplet of the sample was added to the glow discharged grids, and blotted away using filter paper (Whatman no.4) for 3 seconds at 95-100% humidity and room temperature, using an EM GP (Leica). The grid was subsequently plunged into liquid ethane/propane (2:1) at -196 °C. Grids were transferred into a Talos Arctica (Thermo Fisher Scientific) and images were acquired using EPU (Thermo Fisher Scientific) in multi-grid mode, at 0.55 nm/pixel, 15000x nominal magnification. Images were recorded on a K3 direct electron detector (Gatan) in counting mode and ZLP imaging in movie mode, a defocus of -5 micron, and an electron dose of ~4 e/A2/s with 8 seconds exposure time (corresponding to a total dose of 35 e/A2). Movies (80 frames in total) were aligned using MotionCor2 (RRID:SCR_016499) and converted to tiff using EMAN2.

**Nonidet P-40 (NP40) detergent does not interfere with the binding capabilities of rVAR2 to chondroitin sulfate (CS)**

Figure S5 shows the rVAR2 binding to recombinant decorin in the presence of different concentrations of NP40. NP40 is used as a detergent control to lyse extracellular vesicles. Therefore, we tested whether NP40 breaks the rVAR2 CSA attached to recombinant decorin in an ELISA - CSA Binding assay. NP40 does not interfere with rVAR2 binding, supporting that the effect we observe after NP40 addition to EV samples is caused by EV lysis and not by a direct effect on rVAR2 binding.

**Generation of C9 antibody fragments binding ofCS and EV labeling** C9 single chain variable fragment (scFv) was generated using a phage display system from the naïve LiAb-SFMAX^TM^ library, as previously described.^3^ The ofCS specificity of C9 scFv was validated by ELISA, screening for binding to ofCS-modified proteoglycans (ofCSPG), and counter-screening using chondroitinase-treated ofCSPG and heparan sulfate-modified proteoglycans (HSPG).

C9 scFv and Alexa 647-conjugated SpyCatcher dimer were mixed at a 1.8:1 molar ratio and incubated for 1 hour at RT in the dark. To remove potential C9-AF647 aggregates, the mixture was centrifuged at 19000 x g for 5 minutes at 4°C. The supernatant was then collected, leaving 10-15 µl behind in the tube. EV samples were prepared as described in the Methods section. Samples were labeled with 800 nM C9-AF647 dimer and 2 µg/mL CD81-AF488 and incubated for 2 hours at RT in the dark. Thereafter, samples, including negative controls, were kept on ice in the dark until further processing and analysis, as described in the Methods section.

**References**

1. Moon, M.J., Rai, A., Sharma, P., Fang, H., McFadyen, J.D., Greening, D.W., and Peter, K. (2024). Differential effects of physiological agonists on the proteome of platelet-derived extracellular vesicles. Proteomics. https://doi.org/10.1002/PMIC.202300391.

2. Wolf, M., Poupardin, R.W., Ebner-Peking, P., Andrade, A.C., Blöchl, C., Obermayer, A., Gomes, F.G., Vari, B., Maeding, N., Eminger, E., et al. (2022). A functional corona around extracellular vesicles enhances angiogenesis, skin regeneration and immunomodulation. J Extracell Vesicles *11*, e12207. https://doi.org/10.1002/JEV2.12207.

3. Vidal-Calvo, E.E., Martin-Salazar, A., Choudhary, S., Dagil, R., Raghavan, S.S.R., Duvnjak, L., Nordmaj, M.A., Clausen, T.M., Skafte, A., Oberkofler, J., et al. (2024). Tumor-agnostic cancer therapy using antibodies targeting oncofetal chondroitin sulfate. Nature Communications 2024 15:1 *15*, 1–20. https://doi.org/10.1038/s41467-024-51781-0.
